# Measuring guideline adherence in physiotherapy: A scoping review of methodological approaches

**DOI:** 10.1111/jep.14218

**Published:** 2024-10-27

**Authors:** Carolin Bahns, Bettina Scheffler, Alexander Bremer, Christian Kopkow

**Affiliations:** ^1^ Department of Therapy Science I Brandenburg University of Technology Cottbus‐Senftenberg Senftenberg Germany

**Keywords:** adherence to guidelines, clinical practice guideline, measurement, operationalisation, physical therapy

## Abstract

**Rationale:**

Clinical practice guidelines summarise the existing evidence on specific health conditions and aim to optimise quality of care by providing evidence‐based recommendations. Studies have reported a gap between research findings and clinical practice in physiotherapy. Guideline adherence is often used as a measure of agreement between therapeutic care and guideline recommendations. However, there is currently no standardised methodological approach for measuring guideline adherence.

**Aims and Objective:**

The objective of this scoping review was to summarise the methods and results of studies that assessed guideline adherence in physiotherapy.

**Methods:**

MEDLINE, EMBASE, PEDro and CENTRAL databases were searched for relevant literature up to December 2022. Published reports of observational studies and controlled clinical trials that provided information on the assessment of guideline adherence in physiotherapists were included. The selection process was performed independently by two reviewers. The methodological quality of the identified reports was not assessed. Results were summarised narratively.

**Results:**

From a total of 2560 potentially relevant records, 53 reports were included in the analysis. Physiotherapists' adherence to guidelines was primarily assessed in the context of musculoskeletal conditions, such as low back pain (*n* = 25, 47.2%) and osteoarthritis (*n* = 8, 15.1%). A wide range of measurement approaches were used with the majority of reports using web‐based surveys (*n* = 21, 39.6%), followed by chart reviews (*n* = 17, 32.1%). Most reports (*n* = 21, 39.6%) provided information on the level of adherence in terms of frequency dichotomising (self‐reported) clinical practice as adherent or non‐adherent. Adherence rates varied widely between included reports.

**Conclusions:**

Although the large number of included reports indicates a high level of interest in the topic of guideline adherence, there is considerable heterogeneity between studies regarding the methodological approaches used to assess guideline adherence in physiotherapists. This reduces the comparability of the study results.

**Trial Registration:**

INPLASY (registration no. 202250081). Registered on 12th May 2022.

**Abbreviation**
MeSH: Medical Subject Heading

## 1 INTRODUCTION

Clinical practice guidelines systematically summarise the best available evidence on the management of specific health conditions and provide evidence‐based recommendations to assist healthcare professionals and patients in making clinical decisions to optimise the quality of care.^1^ Several studies have shown that physiotherapy delivered according to current guidelines can lead to improved patient outcomes (e.g. pain, function, quality of life), while contributing to reduced utilisation of medical services and lower healthcare costs.^2^, ^3^, ^4^, ^5^ However, many physiotherapists appear not to adhere to guidelines. Instead of providing recommended treatments, they often choose treatments that are not recommended or have conflicting evidence.^6^

Guideline adherence was indexed as a Medical Subject Heading (MeSH) in PubMed in 1998 and defined as ‘conformity in fulfilling or following official, recognised, or institutional requirements, guidelines, recommendations, protocols, pathways, or other standards’.^7^ While ‘adherence’ is the term most commonly used in the literature, ‘conformity’, ‘compliance’ or ‘concordance’ are often used as synonyms. Adherence rates can contribute to quantify the quality of patient care, evaluate health services and providers, optimise guidelines, and support implementation research.^8^, ^9^ Findings on adherence can detect short‐term changes in healthcare providers' attitudes and behaviour, whereas changes in patient‐relevant outcomes often take longer to become visible.^9^ However, measuring guideline adherence is complex and difficult to dichotomise, as the clinical behaviour of healthcare professionals may deviate from recommendations in different ways.^8^ To date, there is little methodological guidance available on how to measure and operationalise guideline adherence, although many different approaches are used which limit the credibility and comparability of results.^8^, ^9^

In 2012, Kolman^10^ conducted a systematic review to summarise different approaches for assessing physiotherapists' adherence to guidelines and nonevidence‐based protocols. The analysed reports showed a high degree of heterogeneity in the measurement and operationalisation of adherence, resulting in rates ranging from 1% to 100%.^10^ Over the last decade, there has been growing interest in evaluating the uptake of guideline recommendations by physiotherapists in clinical practice and new data has emerged.^6^ A scoping review can identify the key components of measuring adherence, highlight limitations and heterogeneity, and provide direction for future research in this field.

The objective of this scoping review was to summarise the methods and results of studies that assessed guideline adherence in physiotherapy.

## 2 METHODS

This scoping review was reported in accordance with the criteria of the Preferred Reporting Items for Systematic Reviews and Meta‐Analyses Extension for Scoping Reviews (PRISMA‐ScR) checklist.^11^ The study protocol was registered prospectively with INPLASY (registration number INPLASY202250081) in May 2022. The study was initially intended to be a systematic review, but the design was changed to a scoping review, as scoping reviews are more exploratory in nature and more suitable for investigating how research has been conducted.^12^ As a result, the deviations from the protocol included: (a) no quality assessment, and (b) narrative description of clinical and methodological factors that might explain heterogeneity without performing statistical analysis.

### 2.1 Search strategy

A comprehensive literature search was performed in MEDLINE (via Ovid), EMBASE (via Ovid), PEDro and CENTRAL databases up to December 2022. The search strategy combined MeSH terms and related terms for ‘physiotherapy’, ‘guideline’ and ‘adherence’ and was modified accordingly for each database. No date or language restrictions were applied. Details of the search strategy are available in Additional file S1. In addition, the reference lists of the included reports and related key articles^2^, ^6^, ^10^ were manually reviewed by the first author (CB) to supplement the electronic search.

### 2.2 Eligibility criteria

This scoping review focused on studies that assessed guideline adherence among physiotherapists regarding their clinical practice. Reports of cross‐sectional studies, cohort studies, case‐control studies and controlled clinical trials published in English or German language were eligible for inclusion. Studies that examined adherence among a mixed sample of healthcare professionals were included if more than 50% of participants were physiotherapists or if data for physiotherapists were presented separately. Studies focusing on physiotherapy students were excluded. To identify a wide range of different approaches used in the literature, any study that compared (self‐reported) clinical practice with guideline recommendations with the aim of assessing the quality of patient care or evaluating the effectiveness of implementation interventions was considered eligible for inclusion. Results on therapists' knowledge of or agreement with guidelines were not taken into account. Adherence had to be assessed against clinical practice guidelines that were developed by government agencies at all levels, institutions, organisations such as professional societies or governing boards, or by convening of expert panels. Studies that assessed adherence using quality indicators developed on the basis of clinical practice guidelines were also included. The underlying guidelines had to be referenced in the abstract, the aim or objective of the study, the methods section or in a published study protocol or register entry, and recommendations from the guidelines had to be listed. Studies assessing adherence to other decision aids (e.g. clinical pathways) were excluded. Only guidelines focusing on the management of specific clinical conditions were included. Studies investigating adherence to hygiene guidelines, safety protocols or other nonclinical work processes were not taken into account.

### 2.3 Selection process

Citations from identified records were imported into EndNote 20 software (Clarivate Analytics, USA) and duplicates were automatically and manually removed. Two reviewers (CB and BS) independently screened titles and abstracts for eligibility based on the predefined inclusion/exclusion criteria using Rayyan QCRI, an open‐source web tool developed to assist researchers in conducting systematic reviews.^13^ Agreement between the two reviewers was calculated using the Cohen's kappa coefficient. Subsequently, the full‐texts of the remaining reports were obtained and also reviewed independently by two reviewers (CB, AB and CK). Disagreements were resolved through discussion. The reasons for exclusion of full‐text reports were documented.

### 2.4 Data extraction and synthesis

Data on the main characteristics of the identified reports, including first author, year of publication, country of origin, study design, health condition, population and sample size, setting, number of guidelines, and context of care, were extracted using a predefined data extraction form. To describe the methods used to assess guideline adherence, information on the methods of assessment, unit of analysis, definition, and quantification were recorded. Where available, the reported rate of guideline adherence was extracted. For reports of interventional or longitudinal studies, only baseline data and/or results from the control group were considered. Data extraction was performed by the first author (CB) and a sample of 10% of the reports were independently checked for accuracy by a second reviewer (AB). Results were presented in tables and summarised narratively. The methodological quality of the included reports was not assessed.

## 3 RESULTS

### 3.1 Literature search

**Figure 1:**
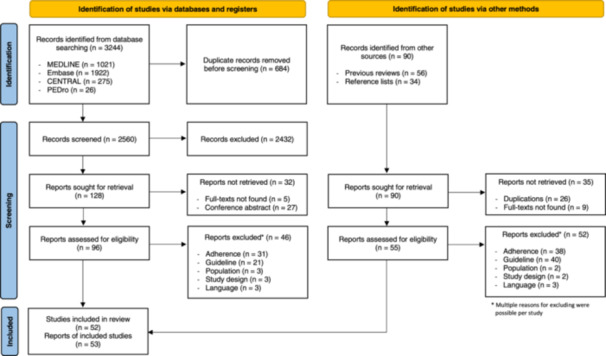
PRISMA (Preferred Reporting Items for Systematic Reviews and Meta‐Analysis)^65^ flow chart.

The database search yielded 3244 records. After removing 684 duplicates, 2560 titles and abstracts were screened for eligibility. There was substantial agreement between both reviewers in the selection of appropriate reports at title and abstract level, given a Cohen's kappa value of 0.716. A full‐text review of 151 potentially relevant reports was performed, including 55 additional reports identified by hand searching. A total of 53^3^, ^4^, ^14^, ^15^, ^16^, ^17^, ^18^, ^19^, ^20^, ^21^, ^22^, ^23^, ^24^, ^25^, ^26^, ^27^, ^28^, ^29^, ^30^, ^31^, ^32^, ^33^, ^34^, ^35^, ^36^, ^37^, ^38^, ^39^, ^40^, ^41^, ^42^, ^43^, ^44^, ^45^, ^46^, ^47^, ^48^, ^49^, ^50^, ^51^, ^52^, ^53^, ^54^, ^55^, ^56^, ^57^, ^58^, ^59^, ^60^, ^61^, ^62^, ^63^, ^64^ reports based on 52 different studies met the inclusion criteria and were included in the analysis. The detailed selection process is summarised in Figure 1. A list of the excluded reports and the reasons for exclusion is provided in Additional file 2.

### 3.2 Study characteristics

Of the 53 reports included in the review, 24 (45.3%) had a cross‐sectional design,^14^, ^15^, ^16^, ^17^, ^18^, ^19^, ^20^, ^21^, ^22^, ^23^, ^24^, ^25^, ^26^, ^27^, ^28^, ^29^, ^30^, ^31^, ^32^, ^33^, ^34^, ^35^, ^36^, ^37^ 16 (30.2%) were based on cohort studies,^3^, ^4^, ^38^, ^39^, ^40^, ^41^, ^42^, ^43^, ^44^, ^45^, ^46^, ^47^, ^48^, ^49^, ^50^, ^51^ one (1.9%) on a case‐control study^52^ and 12 (22.6%) on clinical trials.^53^, ^54^, ^55^, ^56^, ^57^, ^58^, ^59^, ^60^, ^61^, ^62^, ^63^, ^64^ The reports came from 15 different countries, with the majority coming from Europe (*n* = 31, 58.5%).^3^, ^16^, ^17^, ^18^, ^19^, ^20^, ^21^, ^22^, ^24^, ^26^, ^30^, ^32^, ^34^, ^35^, ^36^, ^37^, ^40^, ^42^, ^46^, ^48^, ^49^, ^51^, ^53^, ^55^, ^56^, ^57^, ^58^, ^59^, ^61^, ^62^, ^63^ The Netherlands demonstrated the greatest interest in research on physiotherapy guideline adherence, with 15 reports (28.3%) coming from this country.^3^, ^30^, ^32^, ^35^, ^40^, ^42^, ^46^, ^49^, ^51^, ^53^, ^55^, ^56^, ^57^, ^59^, ^63^ The reports included in this review were published between 2005^48^, ^49^, ^53^ and 2022.^14^, ^15^, ^18^, ^21^, ^22^, ^31^, ^33^, ^36^, ^45^, ^52^, ^54^, ^60^ Almost half of the reports (*n* = 25, 47.2%) assessed guideline adherence in the field of low back pain.^3^, ^15^, ^17^, ^20^, ^23^, ^25^, ^26^, ^27^, ^28^, ^29^, ^31^, ^35^, ^38^, ^39^, ^43^, ^48^, ^49^, ^52^, ^53^, ^54^, ^58^, ^59^, ^61^, ^62^, ^63^ A total of 24 reports (45.3%) focused on physiotherapists working in an outpatient setting only.^3^, ^4^, ^15^, ^16^, ^19^, ^22^, ^27^, ^35^, ^38^, ^42^, ^43^, ^45^, ^46^, ^48^, ^49^, ^50^, ^52^, ^53^, ^54^, ^58^, ^60^, ^61^, ^62^, ^63^ The median number of guidelines from which recommendations were derived as a basis for evaluating adherence was 1, with the number varying between 1 and 12. The majority of reports assessed adherence regarding treatment options (*n* = 48, 90.6%),^3^, ^4^, ^14^, ^15^, ^16^, ^17^, ^18^, ^19^, ^20^, ^21^, ^22^, ^23^, ^24^, ^25^, ^26^, ^28^, ^29^, ^30^, ^31^, ^32^, ^33^, ^34^, ^35^, ^36^, ^37^, ^38^, ^39^, ^40^, ^41^, ^42^, ^44^, ^45^, ^46^, ^47^, ^48^, ^49^, ^50^, ^51^, ^52^, ^53^, ^54^, ^55^, ^57^, ^58^, ^59^, ^60^, ^62^, ^63^ whereas assessment aspects were investigated less often (*n* = 16, 30.2%).^3^, ^17^, ^18^, ^24^, ^35^, ^40^, ^44^, ^46^, ^47^, ^51^, ^55^, ^56^, ^57^, ^59^, ^62^, ^63^ Management aspects such as referrals to specialists or screening red flags were assessed in 29 reports (54.7%).^3^, ^18^, ^21^, ^23^, ^24^, ^27^, ^29^, ^30^, ^31^, ^34^, ^35^, ^42^, ^43^, ^46^, ^47^, ^48^, ^49^, ^51^, ^53^, ^54^, ^55^, ^56^, ^57^, ^58^, ^59^, ^61^, ^62^, ^63^, ^64^ A summary of the key characteristics, including study design, country of origin, and health condition of the included reports is presented in Table 1. Further details for each individual report can be found in Additional file 3.

**Table 1:** Summary of key characteristics of included reports (*n* = 53).

	*n* (%)
Study design	
Cross‐sectional	24 (45.3)
Cohort	16 (30.2)
Case‐control	1 (1.9)
RCT	7 (13.2)
Non‐randomised intervention study	5 (9.4)
Country	
Europe	
The Netherlands	15 (28.3)
UK	4 (7.5)
Sweden	3 (5.7)
France	2 (3.8)
Germany	2 (3.8)
Italy	2 (3.8)
Belgium	1 (1.9)
Denmark	1 (1.9)
Ireland	1 (1.9)
Australia/Oceania	
Australia	8 (15.1)
New Zealand	2 (3.8)
North America	
USA	8 (15.1)
South America	
Brazil	2 (3.8)
Asia	
Saudi Arabia	1 (1.9)
Africa	
Ghana	1 (1.9)
Health condition	
Low back pain	25 (47.2)
Hip/knee osteoarthritis	8 (15.1)
Ankle injuries	4 (7.5)
Stroke	3 (5.7)
Neck pain	2 (3.8)
Upper extremity complaints	2 (3.8)
Arthritis	1 (1.9)
Spinal pain	1 (1.9)
Total hip and knee arthroplasty	1 (1.9)
Whiplash	1 (1.9)
Vertigo	1 (1.9)
Patellofemoral pain	1 (1.9)
Risk of falls	1 (1.9)
Mixed	2 (3.8)
Setting	
Outpatient	24 (45.3)
Inpatient	3 (5.7)
Mixed	17 (32.1)
Not reported	9 (17.0)
Number of guidelines, *median (range)*	1 (1‐12)
Context of care^a^	
Assessment	16 (30.2)
Treatment	48 (90.6)
Management	29 (54.7)

^a^ more than one category possible per study.

### 3.3 Assessment of guideline adherence

#### 3.3.1 Methods

The most frequently used term to describe the agreement between clinical practice and guideline recommendations was *adherence* (*n* = 37, 69.8%),^3^, ^4^, ^16^, ^17^, ^18^, ^21^, ^23^, ^24^, ^25^, ^26^, ^27^, ^28^, ^29^, ^31^, ^35^, ^36^, ^37^, ^38^, ^39^, ^40^, ^42^, ^43^, ^44^, ^46^, ^49^, ^50^, ^51^, ^52^, ^53^, ^54^, ^55^, ^56^, ^57^, ^58^, ^59^, ^63^, ^64^ followed by *compliance* (*n* = 11, 20.8%).^22^, ^24^, ^27^, ^30^, ^33^, ^42^, ^46^, ^47^, ^48^, ^61^, ^62^ Four reports (7.5%) used both terms interchangeably.^24^, ^27^, ^42^, ^46^ The terms *alignment* (*n* = 2, 3.8%)^41^, ^45^ and *concordance* (*n* = 1, 1.9%)^60^ were not frequently used. Six reports (11.3%) with the objective of comparing clinical practice with guideline recommendations did not provide a specific term for the construct of guideline adherence.^14^, ^15^, ^19^, ^20^, ^32^, ^34^

The majority of reports assessed guideline adherence based on self‐reported clinical practice. Web‐based surveys were conducted in 21 reports (39.6%),^14^, ^15^, ^16^, ^17^, ^18^, ^19^, ^21^, ^23^, ^24^, ^25^, ^26^, ^27^, ^28^, ^29^, ^30^, ^31^, ^32^, ^33^, ^34^, ^36^, ^37^ with 11 of these including clinical vignettes.^14^, ^18^, ^21^, ^23^, ^25^, ^26^, ^27^, ^28^, ^29^, ^31^, ^34^ Three reports (5.7%) collected data with postal surveys,^20^, ^24^, ^35^ two of which used clinical vignettes.^20^, ^35^ Questionnaires were used in six reports (11.3%),^54^, ^55^, ^56^, ^57^, ^59^, ^63^ with four of those reports providing clinical vignettes.^54^, ^55^, ^59^, ^63^ Prospective data collection using standardised recording forms was reported in eight reports (15.1%).^3^, ^22^, ^40^, ^53^, ^58^, ^61^, ^62^, ^66^ In total, 17 reports (32.1%) were based on secondary data from patient records,^41^, ^43^, ^44^, ^45^, ^46^, ^47^, ^48^, ^50^, ^60^, ^64^ registries,^42^, ^58^ databases^4^, ^38^, ^39^, ^49^ and charge codes.^52^ Two reports (3.8%) employed a combination of two different data collection methods.^24^, ^58^ Quality indicators were used in 16 reports (30.2%).^3^, ^35^, ^37^, ^40^, ^42^, ^46^, ^49^, ^51^, ^52^, ^55^, ^56^, ^57^, ^58^, ^59^, ^63^, ^64^

More than half of the included reports (*n* = 30, 56.6%) assessed guideline adherence on an individual basis for each physiotherapist and summed it up across the study population to describe the proportion of guideline‐adherent providers.^14^, ^15^, ^16^, ^17^, ^18^, ^19^, ^20^, ^21^, ^22^, ^23^, ^24^, ^25^, ^26^, ^27^, ^28^, ^29^, ^30^, ^31^, ^32^, ^33^, ^34^, ^36^, ^47^, ^49^, ^52^, ^54^, ^55^, ^56^, ^57^, ^63^ A total of 18 reports (34%) analysed adherence on a patient level, reporting the percentage of patients treated according to evidence‐based recommendations.^4^, ^38^, ^39^, ^42^, ^43^, ^44^, ^45^, ^46^, ^48^, ^49^, ^50^, ^51^, ^53^, ^58^, ^60^, ^61^, ^62^, ^64^ In ten reports (18.9%), adherence was analysed on an action level, with the percentage of guideline‐adherent procedures provided.^3^, ^31^, ^35^, ^36^, ^37^, ^40^, ^41^, ^46^, ^59^ Four reports (7.5%) provided information on guideline adherence for more than one unit of analysis.^31^, ^36^, ^46^, ^49^

Approaches to operationalising guideline adherence varied widely, and only a few reports used validated measures or referred to the methods used in previous studies. While many reports used only positive recommendations to define adherence, others also included recommendations against screening or treatment options. Several reports assessed adherence to individual selected recommendations, whereas others assessed adherence to all recommendations relevant to physiotherapy. In certain cases, adherence was defined as performing all recommended behaviours. In contrast, some reports set cut‐off values or a minimum number of expected behaviours, allowing more flexibility to deviate from recommendations. Adherence was quantified using a binary (e.g. adherent/non‐adherent),^4^, ^17^, ^22^, ^27^, ^28^, ^29^, ^31^, ^33^, ^36^, ^37^, ^38^, ^39^, ^40^, ^42^, ^47^, ^49^, ^51^, ^52^, ^53^, ^58^ multi‐categorical (e.g. fully adherent/partially adherent/non‐adherent)^18^, ^20^, ^21^, ^23^, ^25^, ^26^, ^31^, ^46^, ^60^ or metric measure.^3^, ^35^, ^46^, ^52^, ^55^, ^56^, ^57^, ^59^, ^63^ In total, 18 reports (34%) did not operationalise adherence and only reported the use of guideline recommendations at item level.^14^, ^15^, ^16^, ^19^, ^24^, ^30^, ^32^, ^34^, ^41^, ^43^, ^44^, ^45^, ^48^, ^50^, ^54^, ^61^, ^62^, ^64^ A summary of the methods used to assess guideline adherence is presented in Table 2. Further details for each individual report can be found in Additional file 4.

**Table 2:** Methods to assess guideline adherence.

	*n* (%)
Synonym^a^	
Adherence	37 (69.8)
Compliance	11 (20.8)
Alignment	2 (3.8)
Concordance	1 (1.9)
Not reported	6 (11.3)
Assessment method^a^	
Web‐based survey	21 (39.6)
Postal survey	3 (5.7)
(Electronic) questionnaire	6 (11.3)
Standardised recording forms	8 (15.1)
Chart review	17 (32.1)
National database	4 (7.5)
Electronic register	2 (3.8)
Clinical patient records	10 (18.9)
Charge codes	1 (1.9)
Unit of analysis^a^	
Provider	30 (56.6)
Patient	18 (34.0)
Action	10 (18.9)
Operationalisation (quantification)^a^	
Binary measure	21 (39.6)
Multi‐categorical measure	9 (17.0)
Metric measure	9 (17.0)
No operationalisation	18 (34.0)

^a^ more than one category possible per study.

#### 3.3.2 Adherence rates

Due to the heterogeneous methods of assessing guideline adherence within each report, it is not possible to adequately summarise or compare the adherence rates. Details of the results of the individual reports are provided in Additional file 3.

## 4 DISCUSSION

The objective of this scoping review was to provide a comprehensive overview of the methodological approaches reported in published studies to assess guideline adherence in physiotherapy. A considerable number of reports was identified that assessed adherence to guidelines to describe the quality of clinical practice or to evaluate the effectiveness of implementation interventions. The included reports used a wide range of different data collection tools and ways to operationalise guideline adherence, which limited the comparability of results.

In 2012, Kolman^10^ concluded from 43 reports evaluating physiotherapists' adherence to clinical practice guidelines and nonevidence‐based protocols that there was no standardised approach to assessing adherence and that results were not comparable, with adherence rates ranging from 1% to 100%. Our systematic literature searches up to 2022 identified 46 additional reports, indicating that interest in guideline adherence remains high. However, although some reports employed validated measures or referenced methods of previous studies, many authors still created their own methods to assess adherence. This is also in line with the findings of a scoping review conducted by Kentenich et al.,^67^ which identified a considerable degree of heterogeneity in data sources and collection tools, the definition of guideline adherence, the assignment of recommendation classes and the results in studies focusing on physicians' management for patients with chronic coronary artery disease. In addition, the study indicates that standardised assessment of adherence is challenging across all healthcare professions.^67^

The selection of data sources and collection tools is often determined by the objective of the study and the resources available, and may contribute to a potential over‐ or underestimation of guideline adherence. The majority of reports identified in this review were based on self‐reported data. Surveys are a cost‐effective method for collecting data from a large sample size, but there is a high risk of over‐reporting recommended behaviour, mainly due to socially desirable responding.^8^, ^9^ Guideline adherence may be overestimated by approximately 27% when assessed through self‐report measures, in comparison to objective methods.^68^ To improve comparability between participants, many of the included survey or questionnaire studies used clinical vignettes (*n* = 17, 32.1%). Vignettes simulate clinical scenarios with realistic clinical details and may be presented to clinicians to assess their ability to evaluate, diagnose and treat specific medical conditions.^69^ Vignettes that provide an adequate case‐mix may have acceptable validity and produce more accurate results than those obtained by abstracting relevant information from medical records.^69^, ^70^ However, Brunner et al.^71^ identified a limited concordance between self‐reported therapeutic recommendations measured with clinical vignettes and advice given to similar patients in real practice. The authors concluded that written case scenarios are limited in their ability to simulate the full complexity of the therapist–patient interaction in real clinical practice.^71^ Approximately one‐third of the included reports (*n* = 17, 32.1%) used reviews of medical records, registries or databases to assess guideline adherence among physiotherapists. Data collection is also easy and inexpensive, but is highly dependent on the quality of documentation and often lacks relevant details.^8^, ^9^ Notably, none of the included reports used direct observation, which is considered the gold standard for measuring adherence.^8^ One potential explanation for this may be that this form of data collection is highly time‐ and resource‐consuming.^8^, ^9^ In addition, there is a potential for bias, as physiotherapists may behave in a way that deviates from their typical clinical practice due to awareness of being observed (Hawthorne effect).^8^ Other known methods of assessing adherence, such as qualitative interviews or group discussions, and unannounced visits of standardised patients (individuals trained to act like real patients and simulate a set of symptoms or problems) were also not used. Only two reports (3.8%) used more than one data source to assess adherence. However, these were employed to complement each other, rather than to validate the results. Triangulation of different approaches could help to ensure the reliability of the results, as each method has its own advantages and disadvantages.

Another important factor influencing the observed rates of guideline adherence in studies is the way adherence is operationalised. While some of the included reports used only strong recommendations to define adherence, others included elements with a lower level of recommendation. Most of the included reports dichotomised therapists' behaviour or patient care as adherent or non‐adherent. While some reports defined adherence as the presence of all (strongly) recommended items, others permitted some degree of deviation from the guideline recommendations. For example, physiotherapists who demonstrated adherence to more than 75%^31^, ^52^ or 80%^17^ of recommendations, or cases where patents received more than 75% of recommended treatments^4^, ^38^, ^39^ were classified as adherent. It seems to be not realistic to expect full guideline adherence in clinical practice. In addition to therapists' lack of awareness or rejection of the guidelines, there are several reasons why the recommendations are not always followed. Deviations may occur because some recommendations are only applicable in certain circumstances or to individual patients.^8^ In some cases, it may also be more appropriate to deviate from established guidelines to meet the specific needs of the patient. For example, if a patient presents with personal preferences or individual clinical factors such as comorbidities that are not adequately addressed by the guidelines, clinicians may choose to prioritise individualised treatment over strict adherence to guideline recommendations. The principle of evidence‐based practice emphasises that clinical decisions should be based on the best available evidence, clinical expertise, and patient preferences.^72^, ^73^ By integrating these elements, clinicians can make informed decisions that balance adherence to guidelines with the need for individualised care. However, there is no evidence to support these cut‐off points, and it remains unclear which percentage of adherence represents appropriate care.

In addition to the source of data collection and the operationalisation, several other factors, such as the choice of the underlying guidelines, can influence the measured guideline adherence. The majority of the included reports assessed adherence based on a single specific guideline. However, as there are numerous guidelines for the same health condition with varying complexity, and the recommendations are often not consistent, the adherence rates for the same condition may vary. For example, guidelines for hip and knee osteoarthritis consistently recommend exercise, education, and weight management, but recommendations for other physiotherapy‐related treatments, such as manual therapy or electrotherapy, are sometimes contradictory.^74^ In addition, as new research is generated over time and recommendations may be revised, the time of development and the temporal consistency of guidelines may also be an influencing factor. Standardised sets of quality indicators, defined as ‘measurable elements of practice performance for which there is evidence or consensus that they can be used to assess the quality, and thus change the quality, of care provided’,^75^ can help to overcome these problems. As used in some of the included reports (*n* = 16, 30.2%), they are preferably developed systematically from more than one guideline. However, it is important to note that the competencies and skills of physiotherapists may vary across different contexts and countries. Consequently, the applicability of certain quality indicators may differ depending on the specific setting. In general, individual characteristics of the study population, such as age and sex, but also familiarity with established guidelines, can explain the heterogeneity of study results. External factors, such as incentives, if the study was conducted to evaluate the effectiveness of an implementation intervention, can also influence the results.^67^ In conclusion, it is very important to be aware of the various reasons for heterogeneity, due to study methodology and other factors, and to take them into account when interpreting adherence rates.

### 4.1 Limitations

Only research published in English or German was included, so potentially relevant reports published in other languages may have been overlooked. In addition, the eligibility criteria of this review excluded numerous reports that measured adherence without referring to a specific guideline, which may have resulted in the exclusion of information relevant to our study purpose and objectives. However, this may only have a minor impact on the results, as the total number of included reports is large and the findings clearly indicate a high degree of heterogeneity in approaches used to measure guideline adherence in physiotherapy. Given the lack of a standardised understanding of the concept of guideline adherence and the use of various synonyms and methodological approaches, it was challenging to distinguish reports that measured adherence from those that assessed knowledge of guidelines. The selection process was conducted by two independent reviewers, while the data extraction was performed by a single individual. Only a random sample of 10% of reports was cross‐checked by another reviewer, which may also have introduced a degree of bias into the results. In accordance with the methodology of a scoping review, no quality assessment was conducted on the included papers, as the primary objective of this study was to examine the methods used to assess guideline adherence, rather than to investigate the effects of any intervention. To provide a structured overview of the methodological approaches for assessing guideline adherence, information from other studies^8^, ^9^, ^10^, ^67^ that have addressed this topic was used to develop categories. However, it is possible that some of the methods used in the reports have been simplified to a considerable extent. The categories for data extraction have not been validated.

## 5 CONCLUSION

The findings of this scoping review indicate a high degree of heterogeneity in the assessment of guideline adherence in physiotherapy. The included reports reported a variety of data collection tools and approaches for operationalisation, which limits the comparability of results. A standardised approach to assess guideline adherence is desirable to ensure that study results on guideline adherence are robust, comparable, and applicable in different physiotherapy contexts.

## AUTHOR CONTRIBUTIONS

**Carolin Bahns**: Conceptualisation, literature search, title/abstract screening, full text screening, data extraction and analysis, manuscript drafting; **Bettina Scheffler**: Conceptualisation, title/abstract screening, manuscript feedback; **Alexander Bremer**: Full text screening, data extraction, manuscript feedback; **Christian Kopkow**: Conceptualisation, full text screening, manuscript feedback. All authors read and approved the final manuscript.

## CONFLICT OF INTEREST STATEMENT

The following authors were listed as contributors to one or two of the reviewed reports: Carolin Bahns, Bettina Scheffler and Christian Kopkow. It should be noted that they were not involved in the extraction or analysis of the data presented in these reports. Alexander Bremer has declared that he has no conflicts of interest.

## Supporting information

Supporting information.

Supporting information.

Supporting information.

Supporting information.

## Data Availability

The data that supports the findings of this study are available in the supplementary material of this article.
