# Supplementary material for: Measuring guideline adherence in physiotherapy: A scoping review of methodological approaches
Source: J Eval Clin Pract. 2024 Oct 27;31(5):10.1111/jep.14218. doi: 10.1111/jep.14218 (PMC12381545; doi:10.1111/jep.14218)
Supplement: Supplementary file 1 — Supporting information. [file JEP-31-0-s002.docx]

**Additional file 1:** Search strategy

1. Medline (via Ovid): 02.12.2022

| 1 | exp physical therapy modalities/ or physical therapy specialty/ or physical therapist assistants/ or physical therapists/ or physical therapy department, hospital/ or (physiotherap* or physical therap*).ti,ab. |
| --- | --- |
| 2 | Practice Guidelines as Topic/ or (CPG or (guideline* adj3 (clinical or consensus or practice or evidence-based or evidence based or evidence-informed or evidence informed or recommendation* or management))).ti,ab. |
| 3 | (adhere* or complian* or comply* or accordan* or concordan* or conform* or appropriate* or utili#ation or in line).ti,ab. |
| 4 | Guideline adherence/ |
| 5 | 2 and 3 |
| 6 | 4 or 5 |
| 7 | 1 and 6 |

2. Embase (via Ovid): 02.12.2022

| 1 | physiotherapy/ or physiotherapist/ or physiotherapist assistant/ or (physiotherap* or physical therap*).ticab. |
| --- | --- |
| 2 | Practice guideline/ or (CPG or (guideline* adj3 (clinical or consensus or practice or evidence-based or evidence based or evidence-informed or evidence informed or recommendation* or management))).ti,ab. |
| 3 | (adhere* or complian* or comply* or accordan* or concordan* or conform* or appropriate* or utili?ation or in line).ti,ab. |
|  | Protocol compliance/ |
| 5 | 2 and 3 |
| 6 | 4 or 5 |
| 7 | 1 and 6 |

3. Cochrane Central Register of Controlled Trials (CENTRAL): 02.12.2022

| #1 | (physiotherap* or "physical therapy" or "physical therapist" or "physical therapists"):ti,ab,kw |
| --- | --- |
| #2 | (CPG or (guideline* NEAR/3 (clinical or consensus or practice or evidence-based or "evidence based" or evidence-informed or "evidence informed" or recommendation* or management))):ti,ab,kw |
| #3 | (adhere* or complian* or comply* or accordan* or concordan* or conform* or appropriate* or utili?ation or "in line"):ti,ab,kw |
| #4 | #2 and #3 |
| #5 | #1 and #4 |

4. PEDro: 02.12.2022

| #1 | guideline and adhere* |
| --- | --- |
