# Supplementary material for: Measuring guideline adherence in physiotherapy: A scoping review of methodological approaches
Source: J Eval Clin Pract. 2024 Oct 27;31(5):10.1111/jep.14218. doi: 10.1111/jep.14218 (PMC12381545; doi:10.1111/jep.14218)
Supplement: Supplementary file 2 — Supporting information. [file JEP-31-0-s003.docx]

**Additional file 2:** List of excluded reports

|  | **Report*** | **Reason for exclusion** |
| --- | --- | --- |
| 1 | Akindele M, Rabiu M, Useh E. Assessment of the awareness, adherence, and barriers to low back pain clinical practice guidelines by practicing physiotherapists in a low-resourced country. Physiotherapy research international: the journal for researchers and clinicians in physical therapy. 2020;25(1):e1811. doi: 10.1002/pri.1811 | Guideline (no specific guideline mentioned) |
| 2 | Akodu AK, Osundiya OC, Ajepe TO, Jegede OM. Management of knee osteoarthritis: Knowledge and adherence to clinical practice guidelines among physiotherapists in selected hospitals in Lagos State, Nigeria. African Journal of Biomedical Research. 2020;23(3):327-33. | Guideline (no specific guideline mentioned); Adherence (no evaluation of adherence) |
| 3 | Alt Murphy M, Bjorkdahl A, Forsberg-Warleby G, Persson CU. Implementation of evidence-based assessment of upper extremity in stroke rehabilitation: From evidence to clinical practice. Journal of rehabilitation medicine. 2021;53(1):jrm00148. doi: 10.2340/16501977-2790 | Guideline (local guideline) |
| 4 | Ayanniyi O, Egwu RF, Adeniyi AF. Physiotherapy management of knee osteoarthritis in Nigeria-A survey of self-reported treatment preferences. Hong Kong Physiotherapy Journal. 2017;36:1-9. doi: 10.1016/j.hkpj.2016.07.002 | Adherence (no evaluation of adherence) |
| 5 | Balachandran S, Lee A, Royse A, Denehy L, El-Ansary D. Upper limb exercise prescription following cardiac surgery via median sternotomy: a web survey. Journal of cardiopulmonary rehabilitation and prevention. 2014;34(6):390-5. doi: 10.1097/HCR.0000000000000053 | Guideline (no specific guideline mentioned); Adherence (no evaluation of adherence) |
| 6 | Beales D, Hope JB, Hoff TS, Sandvik H, Wergeland O, Fary R. Current practice in management of pelvic girdle pain amongst physiotherapists in Norway and Australia. Manual therapy. 2015;20(1):109-16. doi: 10.1016/j.math.2014.07.005 | Guideline (no specific guideline mentioned); Adherence (no evaluation of adherence) |
| 7 | Bekkering GE, Hendriks HJM, van Tulder MW, Koopmanschap MA, Knol DL, Oostendorp RAB, et al. Effectiviteit van een actieve implementatiestrategie van de KNGF-richtlijn 'Lage-rugpijn' (Effectiveness of an active intervention strategy for the implementation of the Dutch physiotherapy guideline on low back pain) [Dutch]. Nederlands Tijdschrift voor Fysiotherapie [Dutch Journal of Physical Therapy] 2005 Jun;115(3):62-67. 2005. | Language |
| 8 | Bernhardsson S, Larsson MEH, Eggertsen R, Olsen MF, Johansson K, Nilsen P, et al. Evaluation of a tailored, multi-component intervention for implementation of evidence-based clinical practice guidelines in primary care physical therapy: a non-randomized controlled trial. BMC health services research. 2014;14:105. doi: 10.1186/1472-6963-14-105 | Guideline (own guideline development); Adherence (no evaluation of adherence) |
| 9 | Bernhardsson S, Larsson MEH. Does a tailored guideline implementation strategy have an impact on clinical physiotherapy practice? A nonrandomized controlled study. Journal of evaluation in clinical practice. 2019;25(4):575-84. doi: 10.1111/jep.12958 | Guideline (own guideline development) |
| 10 | Brindisino F, De Santis A, Rossettini G, Pellicciari L, Filipponi M, Rollo G, et al. Post-surgery rehabilitation following rotator cuff repair. A survey of current (2020) Italian clinical practice. Disability and rehabilitation. 2022;44(17):4689-99. doi: 10.1080/09638288.2021.1916628 | Adherence (no evaluation of adherence) |
| 11 | Carlesso LC, MacDermid JC, Gross AR, Walton DM, Santaguida PL. Treatment preferences amongst physical therapists and chiropractors for the management of neck pain: Results of an international survey. Chiropractic and Manual Therapies. 2014;22(1):11. doi: https: 10.1186/2045-709X-22-11 | Population (no separate data for PT); Guideline (no specific guideline mentioned); Adherence (no evaluation of adherence) |
| 12 | Conradsson D, Leavy B, Hagströmer M, Nilsson MH, Franzén E. Physiotherapy for Parkinson's Disease in Sweden: Provision, Expertise, and Multi-professional Collaborations. Mov Disord Clin Pract. 2017 Aug 20;4(6):843-851. doi: 10.1002/mdc3.12525 | Adherence (no evaluation of adherence) |
| 13 | Corkery MB, Edgar KL, Smith CE. A survey of physical therapists' clinical practice patterns and adherence to clinical guidelines in the management of patients with whiplash associated disorders (WAD). Journal of Manual and Manipulative Therapy. 2014;22(2):75-89. doi: 10.1179/2042618613Y.0000000048 | Adherence (no evaluation of adherence) |
| 14 | Derghazarian T, Simmonds MJ. Management of low back pain by physical therapists in quebec: how are we doing? Physiotherapy Canada Physiotherapie Canada. 2011;63(4):464-73. doi: 10.3138/ptc.2010-04P | Guideline (no specific guideline mentioned); Adherence (no evaluation of adherence) |
| 15 | Duran-Palomino D, Chapeton O, Martinez-Santa J, Campos-Rodriguez A, Ramirez-Velez R. [Adherence to the recommendations in respiratory rehabilitation of the British Thoracic Society in patients with cystic fibrosis: a study of Colombian physiotherapists]. Cumplimiento de las recomendaciones en rehabilitacion respiratoria de la British Thoracic Society en pacientes con fibrosis quistica: estudio en fisioterapeutas colombianos. 2013;30(2):256-61. | Language |
| 16 | Evans DW, Breen AC, Pincus T, Sim J, Underwood M, Vogel S, et al. The effectiveness of a posted information package on the beliefs and behavior of musculoskeletal practitioners: the UK Chiropractors, Osteopaths, and Musculoskeletal Physiotherapists Low Back Pain ManagemENT (COMPLeMENT) randomized trial. Spine. 2010;35(8):858-66. doi: 10.1097/BRS.0b013e3181d4e04b | Population (<50% PTs, no separate data for PTs) |
| 17 | Faletra A, Bellin G, Dunning J, Fernandez-de-Las-Penas C, Pellicciari L, Brindisino F, et al. Assessing cardiovascular parameters and risk factors in physical therapy practice: findings from a cross-sectional national survey and implication for clinical practice. BMC musculoskeletal disorders. 2022;23(1):749. doi: 10.1186/s12891-022-05696-w | Adherence (no evaluation of adherence) |
| 18 | Fidvi N, May S. Physiotherapy management of low back pain in India - a survey of self-reported practice. Physiotherapy research international: the journal for researchers and clinicians in physical therapy. 2010;15(3):150-9. doi: 10.1002/pri.458 | Guideline (no specific guideline mentioned); Adherence (no evaluation of adherence) |
| 19 | Fritz JM, Cleland JA, Brennan GP. Does adherence to the guideline recommendation for active treatments improve the quality of care for patients with acute low back pain delivered by physical therapists? Medical care. 2007;45(10):973-80. | Guideline (no specific guideline mentioned) |
| 20 | Fritz JM, Cleland JA, Speckman M, Brennan GP, Hunter SJ. Physical therapy for acute low back pain: associations with subsequent healthcare costs. Spine. 2008;33(16):1800-5. doi: 10.1097/BRS.0b013e31817bd853 | Guideline (no specific guideline mentioned) |
| 21 | Guessous I, Cornuz J, Stoianov R, Burnand B, Fitting J-W, Yersin B, et al. Efficacy of clinical guideline implementation to improve the appropriateness of chest physiotherapy prescription among inpatients with community-acquired pneumonia. Respiratory medicine. 2008;102(9):1257-63. doi: 10.1016/j.rmed.2008.04.008 | Adherence (evaluation of prescription rate) |
| 22 | Hammond R, Lennon S, Walker MF, Hoffman A, Irwin P, Lowe D, et al. Changing occupational therapy and physiotherapy practice through guidelines and audit in the United Kingdom. Clinical rehabilitation. 2005;19(4):365-71. | Adherence (no evaluation of adherence) |
| 23 | Holden MA, Bennell KL, Whittle R, Chesterton L, Foster NE, Halliday NA, et al. How Do Physical Therapists in the United Kingdom Manage Patients With Hip Osteoarthritis? Results of a Cross-Sectional Survey. Physical therapy. 2018;98(6):461-70. doi: 10.1093/ptj/pzy013 | Adherence (no evaluation of adherence) |
| 24 | Holden MA, Nicholls EE, Hay EM, Foster NE. Physical therapists' use of therapeutic exercise for patients with clinical knee osteoarthritis in the United kingdom: in line with current recommendations? Physical therapy. 2008;88(10):1109-21. doi: 10.2522/ptj.20080077 | Guideline (exercise recommendations) |
| 25 | Hurkmans EJ, Li L, Verhoef J, Vliet Vlieland TPM. Physical therapists' management of rheumatoid arthritis: results of a Dutch survey. Musculoskeletal care. 2012;10(3):142-8. doi: 10.1002/msc.1011 | Adherence (no evaluation of adherence) |
| 26 | Hurley N, Kehoe B, McCaffrey N, Redmond K, Cullen L, Moyna NM. Recommendations to improve physical activity prescription for the cystic fibrosis population: an Irish perspective. BMC health services research. 2020;20(1):1052. doi: 10.1186/s12913-020-05910-2 | Guideline (no specific guideline mentioned); Adherence (no evaluation of adherence) |
| 27 | Jolliffe L, Hoffmann T, Churilov L, Lannin NA. What is the feasibility and observed effect of two implementation packages for stroke rehabilitation therapists implementing upper limb guidelines? A cluster controlled feasibility study. BMJ open quality. 2020;9(2). doi: 10.1136/bmjoq-2020-000954 | Population (<50% PTs, no separate data for PTs) |
| 28 | Keen C, Fowler-Davis S, McLean S, Manson J. Physiotherapy practice in pulmonary hypertension: physiotherapist and patient perspectives. Pulmonary Circulation. 2018;8(3). doi: 10.1177/2045894018783738 | Guideline (no specific guideline mentioned); Adherence (no evaluation of adherence) |
| 29 | M S A, Kooven S, Al-Mudahka N. Adherence of physical therapy with clinical practice guidelines for the rehabilitation of stroke in an active inpatient setting. Disability and rehabilitation. 2019;41(15):1855-62. doi: 10.1080/09638288.2018.1449257 | Guideline (local guideline) |
| 30 | Nijkrake MJ, Keus SH, Ewalds H, Overeem S, Braspenning JC, Oostendorp RA, Hendriks EJ, Bloem BR, Munneke M. Quality indicators for physiotherapy in Parkinson's disease. Eur J Phys Rehabil Med. 2009 Jun;45(2):239-45. Epub 2009 Apr 20. | Study design |
| 31 | O'Hanlon E, Kennedy N. Exercise in cancer care in Ireland: a survey of oncology nurses and physiotherapists. European journal of cancer care. 2014;23(5):630-9. doi: 10.1111/ecc.12206 | Guideline (no specific guideline mentioned); Adherence (no evaluation of adherence) |
| 32 | Oostendorp RA, Elvers H, van Trijffel E, Rutten GM, Scholten-Peeters GG, Heijmans M, Hendriks E, Mikolajewska E, De Kooning M, Laekeman M, Nijs J, Roussel N, Samwel H. Has the quality of physiotherapy care in patients with Whiplash-associated disorders (WAD) improved over time? A retrospective study using routinely collected data and quality indicators. Patient Prefer Adherence. 2018 Nov 8;12:2291-2308. doi: 10.2147/PPA.S179808 | Adherence (no evaluation of adherence) |
| 33 | Otterman NM, van der Wees PJ, Bernhardt J, Kwakkel G. Physical therapists' guideline adherence on early mobilization and intensity of practice at dutch acute stroke units: a country-wide survey. Stroke. 2012;43(9):2395-401. doi: 10.1161/STROKEAHA.112.660092 | Adherence (no evaluation of adherence) |
| 34 | Peter WF, van der Wees PJ, Hendriks EJ, de Bie RA, Verhoef J, de Jong Z, van Bodegom-Vos L, Hilberdink WK, Vliet Vlieland TP. Quality indicators for physiotherapy care in hip and knee osteoarthritis: development and clinimetric properties. Musculoskeletal Care. 2013 Dec;11(4):193-202. doi: 10.1002/msc.1041 | Study design |
| 35 | Peters R, Schmitt MA, Verhagen AP, Pool-Goudzwaard AL, Mutsaers JHAM, Koes BW. Comparing the range of musculoskeletal therapies applied by physical therapists with postgraduate qualifications in manual therapy in patients with non-specific neck pain with international guidelines and recommendations: An observational study. Musculoskeletal science & practice. 2020;46:102069. doi: 10.1016/j.msksp.2019.102069 | Adherence (no evaluation of adherence) |
| 36 | Purcell K, Tiedemann A, Kristensen MT, Cunningham C, Hjermundrud V, Ariza-Vega P, et al. Mobilisation and physiotherapy intervention following hip fracture: snapshot survey across six countries from the Fragility Fracture Network Physiotherapy Group. Disability and rehabilitation. 2022;44(22):6788-95. doi: 10.1080/09638288.2021.1974107 | Adherence (no evaluation of adherence) |
| 37 | Rebbeck T, Maher CG, Refshauge KM. Evaluating two implementation strategies for whiplash guidelines in physiotherapy: a cluster randomised trial. Aust J Physiother. 2006;52(3):165-74. doi: 10.1016/s0004-9514(06)70025-3 | Adherence (no evaluation of adherence) |
| 38 | Rutten GM, Harting J, Rutten ST, Bekkering GE, Kremers SP. Measuring physiotherapists' guideline adherence by means of clinical vignettes: a validation study. J Eval Clin Pract. 2006 Oct;12(5):491-500. doi: 10.1111/j.1365-2753.2006.00699.x | Study design |
| 39 | Scalise V, Brindisino F, Pellicciari L, Minnucci S, Bonetti F. Carpal tunnel syndrome: A national survey to monitor knowledge and operating methods. International Journal of Environmental Research and Public Health. 2021;18(4):1-27. doi: 10.3390/ijerph18041995 | Guideline (no specific guideline mentioned); Adherence (no evaluation of adherence) |
| 40 | Schroder K, Oberg B, Enthoven P, Hedevik H, Fors M, Abbott A. Effectiveness and quality of implementing a best practice model of care for low back pain (Betterback) compared with routine care in physiotherapy: A hybrid type 2 trial. Journal of Clinical Medicine. 2021;10(6):1-17. doi: 10.3390/jcm10061230 | Guideline (no specific guideline mentioned) |
| 41 | Smythe A, White J, Littlewood C, Bury J, Haines T, Malliaras P. Physiotherapists deliver management broadly consistent with recommended practice in rotator cuff tendinopathy: An observational study. Musculoskeletal Science and Practice. 2020;47:102132. doi: 10.1016/j.msksp.2020.102132 | Adherence (no evaluation of adherence) |
| 42 | Strand LI, Kvale A, Raheim M, Ljunggren AE. Do Norwegian manual therapists provide management for patients with acute low back pain in accordance with clinical guidelines? Manual therapy. 2005;10(1):38-43. | Guideline (no specific guideline mentioned); Adherence (no evaluation of adherence) |
| 43 | Van Peppen RP, Maissan FJ, Van Genderen FR, Van Dolder R, Van Meeteren NL. Outcome measures in physiotherapy management of patients with stroke: a survey into self-reported use, and barriers to and facilitators for use. Physiother Res Int. 2008 Dec;13(4):255-70. doi: 10.1002/pri.417 | Adherence (no evaluation of adherence) |
| 44 | Walsh NE, Hurley MV. Evidence based guidelines and current practice for physiotherapy management of knee osteoarthritis. Musculoskeletal care. 2009;7(1):45-56. doi: 10.1002/msc.144 | Adherence (no evaluation of adherence) |
| 45 | Werner EL, Indahl A. [Knowledge, practice and attitudes to back pain among doctors, physiotherapists and chiropractors]. Kunnskap, praksis og holdninger til rygglidelser hos leger, fysioterapeuter og kiropraktorer. 2005;125(13):1794-7. | Language |
| 46 | Wittboldt S, Leosdottir M, Ravn Fischer A, Ekman B, Back M. Exercise-based cardiac rehabilitation after acute myocardial infarction in Sweden - standards, costs, and adherence to European guidelines (The Perfect-CR study). Physiotherapy theory and practice. 2022:1-11. doi: 10.1080/09593985.2022.2114052 | Adherence (no evaluation of adherence) |

*reasons for exclusion only reported for reports identified through database search
