# Supplementary material for: Measuring guideline adherence in physiotherapy: A scoping review of methodological approaches
Source: J Eval Clin Pract. 2024 Oct 27;31(5):10.1111/jep.14218. doi: 10.1111/jep.14218 (PMC12381545; doi:10.1111/jep.14218)
Supplement: Supplementary file 3 — Supporting information. [file JEP-31-0-s004.docx]

**Additional file 3:** Characteristics and results of included reports

| **Study** | **Year** | **Country** | **Health condition** | **Population** | **Setting** | **Guidelines** | **Context of care** | **Results reported on item level** | **Adherence rate** |
| --- | --- | --- | --- | --- | --- | --- | --- | --- | --- |
| ***Cross-sectional studies*** | | | | | | | | | |
| Ackah et al. [1] | 2022 | Ghana | Knee osteoarthritis | PTs (N = 148) | Mixed | 1 | Treatment | Yes | NA |
| Ahern et al. [2] | 2022 | Australia | Low back pain | PTs (N = 145),  GPs (N = 55) | Outpatient | 4 | Treatment | Yes | NA |
| Andersson et al. [3] | 2017 | Sweden | Arthritis | PTs (N = 64) | Outpatient | 1 | Treatment | Yes | NA |
| Bahns et al. [4] | 2021 | Germany | Low back pain | PTs (N = 1361) | Mixed | 1 | Assessment, Treatment | Yes | Assessment:  Adherence: 675/1361 (50%)  Non-adherence: 686/1361 (50%)  Treatment:  Adherence: 973/1361 (72%)  Non-adherence: 388/1361 (28%)  Overall:  Adherence: 513/1361 (38%)  Non-adherence: 848/1361 (62%) |
| Battista et al. [5] | 2022 | Italy | Hip/knee osteoarthritis | PTs (N = 822) | NR | 3 | Assessment, Treatment, Management | Yes | Delivering: 202/822 (25%)  Partially delivering: 181/822 (22%)  Non-delivering: 439/822 (53%) |
| Bernhardsson et al. [6] | 2015 | Sweden | Low back pain, neck pain, subacromial pain | PTs (N = 271) | Outpatient | 3 | Treatment | Yes | NA |
| Bishop et al. [7] | 2008 | UK | Low back pain | PTs (N = 580), GPs (N = 442) | Mixed | 4 | Treatment | Yes | Activity  Strictly in line: 132/170 (13%)  Broadly in line: 810/170 (80%)  Not in line: 67/170 (7%)  Work  Strictly in line: 173/994 (17%)  Broadly in line: 542/994 (55%)  Not in line: 279/994 (28%)  Bed rest  Strictly in line: 291/1004 (29%)  Broadly in line: 704/1004 (70%)  Not in line: 9/1004 (1%) |
| Caffini et al. [8] | 2022 | Italy | Lateral ankle sprains | PTs (N = 408) | NR | 3 | Treatment, Management | Yes | Vignette 1:  Following: 17/408 (4%)  Partially following: 278/408 (68%)  Partially not following: 93/408 (23%)  Not following: 20/408 (5%)  Vignette 2:  Following: 151/408 (37%)  Partially following: 143/408 (35%)  Not following: 114/408 (28%) |
| Demont et al. [9] | 2022 | France | Spinal pain | PTs (N = 60), GPs (N = 164), Patients  (N = 300) | Outpatient | 2 | Treatment | Yes | Compliance: 46/60 (77%)  Non-compliance: 14/60 (23%) |
| de Souza et al. [10] | 2017 | Brazil | Low back pain | PTs (N = 189) | Mixed | 1 | Treatment, Management | Yes | Case 1:  Fully adherent: 20/180 (11%)  Partially adherent: 114/180 (63%)  Not adherent: 46/180 (26%)  Case 2:  Fully adherent: 11/171 (6%)  Partially adherent: 72/171 (42%)  Not adherent: 88/171 (52%)  Case 3:  Fully adherent: 9/167 (5%)  Partially adherent: 79/167 (47%)  Not adherent: 79/167 (47%)  Case 4:  Fully adherent: 41/165 (25%)  Partially adherent: NA  Not adherent: 124/165 (75%)  Case 5:  Fully adherent: 24/165 (15%)  Partially adherent: 85/165 (51%)  Not adherent: 56/165 (34%)  Case 6:  Fully adherent:  16/162 (10%)  Partially adherent: 122/162 (75%)  Not adherent: 24/162 (15%) |
| Donohue et al. [11] | 2014 | Ireland | Stroke | PTs (N = 23) | Inpatient | 1 | Assessment, Treatment, Management | Yes | NA |
| Hendrick et al. [12] | 2013 | New Zealand | Low back pain | PTs (N = 170) | Mixed | 4 | Treatment | Yes | Activity  Strictly in line: 60/170 (35%)  Broadly in line: 102/170 (60%)  Not in line: 5/170 (3%)  Work  Strictly in line: 9/170 (5%)  Broadly in line: 149/170 (88%)  Not in line: 12/170 (7%)  Bed rest  Strictly in line: 41/170 (24%)  Broadly in line: 126/170 (63%)  Not in line: - |
| Husted et al. [13] | 2020 | Denmark | Low back pain | PTs (N = 234) | NR | 4 | Treatment | Yes | Vignette 1:  Activity  Strictly in line: 64/201 (32%)  Partly in line: 135/201 (67%)  Not in line: 2/201 (1%)  Work  Strictly in line: 33/201 (16%)  Partly in line: 159/201 (79%)  Not in line: 9/201 (5%)  Psychological risk factors  Strictly in line: 164/201 (82%)  Partly in line: 30/201 (15%)  Not in line: 7/201 (3%)  Vignette 2:  Activity  Strictly in line: 12/198 (6%)  Partly in line: 180/198 (91%)  Not in line: 6/198 (3%)  Work  Strictly in line: 105/198 (53%)  Partly in line: 90/198 (45%)  Not in line: 3/198 (2%)  Psychological risk factors  Strictly in line: 119/198 (60%)  Partly in line: 33/198 (17%)  Not in line: 46/198 (23%) |
| Keating et al. [14] | 2016 | Australia | Low back pain | PTs (N = 203) | Outpatient | 1 | Management | Yes | Vignette 1:  Adherence: 175/197 (89%)  Non-adherence: 22/197 (11%)  Vignette 2:  Adherence: 124/197 (63%)  Non-adherence: 73/197 (37%)  Vignette 3:  Adherence: 124/197 (63%)  Non-adherence: 73/197 (37%)  Vignette 4:  Adherence: 165/197 (84%)  Non-adherence: 32/197 (16%)  Vignette 5:  Adherence: 156/197 (79%)  Non-adherence: 41/197 (21%) |
| Ladeira et al. [15] | 2017 | USA | Low back pain | PTs (N = 410) | Mixed | 1 | Treatment | Yes | Vignette 1:  Adherence: 234/410 (57%)  Non-adherence: 176/410 (43%)  Vignette 2:  Adherence: 189/410 (46%)  Non-adherence: 221/410 (54%)  Vignette 3:  Adherence: 296/410 (72%)  Non-adherence: 114/410 (28%)  Vignette 4:  Adherence: 121/410 (30%)  Non-adherence: 289/410 (70%) |
| Ladeira et al. [16] | 2015 | USA | Low back pain | PTs (N = 327) | Mixed | 12 | Treatment, Management | Yes | Vignette 1:  Adherence: 76/327 (23%)  Non-adherence: 251/327 (77%)  Vignette 2:  Adherence: 108/327 (33%)  Non-adherence: 219/327 (67%) |
| Leemrijse et al. [17] | 2006 | The Netherlands | Acute ankle sprains | PTs (N = 332) | NR | 1 | Treatment, Management | Yes | NA |
| Moslem et al. [18] | 2022 | Saudi Arabia | Low back pain | PTs (N = 133) | Mixed | 1 | Treatment, Management | Yes | Adherence at provider level:  Vignette 1:  Adherence: 14/133 (10%)  Non-adherence: 119/133 (90%)  Vignette 2:  Adherence: 34/117 (29%)  Non-adherence: 83/117 (71%)  Vignette 3:  Adherence: 6/114 (5%)  Non-adherence: 108/114 (95%)  Vignette 4:  Adherence: 18/107 (17%)  Non-adherence: 89/107 (83%)  Vignette 5:  Adherence: 11/104 (11%)  Non-adherence: 93/104 (89%)  Vignette 6:  Adherence: 58/101 (57%)  Non-adherence: 43/101 (43%) |
|  |  |  |  |  |  |  |  |  | Adherence at action level:  High adherence: -  Moderate adherence: 1/6  Fair adherence: 1/6  Low adherence: 4/6 |
| Peter et al. [19] | 2014 | The Netherlands | Total hip and knee arthroplasty | PTs (N = 219) | Mixed | 1 | Treatment | Yes | NA |
| Pisani et al. [20] | 2022 | Brazil | Patellofemoral pain | PTs (N = 194) | NR | 1 | Treatment | Yes | High compliance: 50/194 (26%)  Low compliance: 144/194 (74%) |
| Riera et al. [21] | 2021 | France | Rotator cuff related shoulder pain | PTs (N = 206) | Mixed | 4 | Treatment, Management | Yes | NA |
| Rutten et al. [22] | 2009 | The Netherlands | Low back pain | PTs (N = 472) | Outpatient | 2 | Assessment, Treatment, Management | No | Mean percentage of adherence: 50.4% |
| Scheffler et al. [23] | 2022 | Germany | Stroke | PTs (N = 170) | Mixed | 1 | Treatment | Yes | Adherence at action level:  Excellent adherence: 1/49 (2%) |
| Spitaels et al. [24] | 2017 | Belgium | Knee osteoarthritis | PTs (N = 284) | Mixed | 8 | Treatment | Yes | Good adherence: 6/15 (40%)  Low adherence: 3/15 (20%) |
| ***Cohort studies*** | | | | | | | | | |
| Childs et al. [25] | 2015 | USA | Low back pain | Patients (N = 753,450) | Outpatient | 1 | Treatment | No | Adherence: 30,917/71,559 (43%)  Non-adherence: 40,462/71,559 (57%) |
| Fritz et al. [26] | 2012 | USA | Low back pain | Patients (N = 32,070) | NR | 1 | Treatment | No | Adherence: 413/1917 (22%)  Non-adherence: 1504/1917 (78%) |
| Horn et al. [27] | 2015 | USA | Neck pain | Patients (N = 298) | Outpatient | 1 | Treatment | No | Adherence: 33/298 (11%)  Non-adherence: 265/298 (89%) |
| Jansen et al. [28] | 2010 | The Netherlands | Hip and knee osteoarthritis | PTs (N = 27), Patients (N = 103) | NR | 1 | Assessment, Treatment | Yes | Diagnostic process:  Good adherence: 4/5 (80%)  Therapeutic process:  Good adherence: 3/6 (50%) |
| Johnston et al. [29] | 2013 | New Zealand | Stroke | Patients (N = 94) | Inpatient | 1 | Treatment | Yes | NA |
| Kooijman et al. [30] | 2011 | The Netherlands | Ankle injury & functional instability | PTs (N = 117), Patients (N = 1413) | Outpatient | 1 | Treatment, Management | Yes | Ankle injury:  Adherence: 300/556 (54%)  Non-adherence: 256/556 (46%)  Functional instability:  Adherence: 96/212 (45%)  Non-adherence: 116/212 (55%) |
| Leerar et al. [31] | 2007 | USA | Low back pain | PTs (N = 16), Patients (N = 160) | Outpatient | 1 | Management | Yes | NA |
| Lloyd et al. [32] | 2020 | Australia | Vertigo, dizziness, and imbalance | Patients (N = 96) | Mixed | 1 | Assessment, Treatment | Yes | NA |
| Naylor et al. [33] | 2022 | Australia | Knee osteoarthritis | PTs (N = 15), Patients (N = 26) | Outpatient | 4 | Treatment | Yes | NA |
| Oostendorp et al. [34] | 2013 | The Netherlands | Neck pain | PTs (N = 38), Patients (N = 96) | Outpatient | 5 | Assessment, Treatment, Management | Yes | Adherence at action level:  Mean overall adherence: 55.6% |
|  |  |  |  |  |  |  |  |  | Adherence at patient level:  Negligible adherence: 0/96 (0%)  Low adherence: 0/96 (0%)  Weak adherence: 0/96 (0%)  Very inadequate adherence: 14/96 (15%)  Inadequate adherence: 28/96 (29%)  Adequate/substantial adherence: 52/96 (54%)  Good adherence: 2/96 (2%)  Very good adherence: 0/96 (0%)  Excellent adherence: 0/96 (0%) |
| Rebbeck et al. [35] | 2013 | Australia | Whiplash | PTs (N = 67), Chiropractors (N = 24), Osteopaths (N = 2) | NR | 2 | Assessment, Treatment, Management | Yes | Baseline (PTs only):  Compliance: 42/67 (63%)  Non-compliance: 25/67 (37%) |
| Rutten et al. [36] | 2010 | The Netherlands | Low back pain | PTs (N = 61), Patients (N = 145) | Outpatient | 2 | Assessment, Treatment, Management | Yes | Mean percentage of adherence: 67% (17/25 positive indicators per patients) |
| Sparkes [37] | 2005 | UK | Low back pain | Patients (N = 100) | Outpatient | 1 | Management, Treatment | Yes | NA |
| Swinkels et al. [38] | 2005 | The Netherlands | Low back pain | PTs (N = 90), Patients (N = 1254) | Outpatient | 1 | Treatment, Management | Yes | Adherence at patient level:  Adherence: 660/1254 (53%)  Non-adherence: 594/1254 (47%) |
|  |  |  |  |  |  |  |  |  | Adherence at provider level was not reported in detail |
| Tang et al. [39] | 2020 | Australia | Knee osteoarthritis | Patients (N = 86) | Outpatient | 3 | Treatment | Yes | NA |
| van der Wees et al. [40] | 2007 | The Netherlands | Acute ankle injury | PTs (N = 22), Patients (N = 174) | Mixed | 1 | Assessment, Treatment, Management | Yes | Adherence: 99/174 (57%)  Non-adherence: 75/174 (43%) |
| ***Case-control studies*** | | | | | | | | | |
| Kolb et al. [41] | 2022 | USA | Low back pain | Physiotherapy sites (N = 4) (PTs (N = 17), Physical therapy assistants (N = 13)) | Outpatient | 1 | Treatment | No | Adherence - Baseline results (mean):  Intervention site 1: 70.0%  Intervention site 2: 79.2%  Control site 1: 46.2%  Control site 2: 66.9%  Adherence - Baseline results:  Intervention site 1: 3/18 months  Intervention site 2: 14/18 months  Control site 1: 0/18  Control site 2: 2/18 |
| ***Randomised controlled trials*** | | | | | | | | | |
| Bekkering et al. [42] | 2005 | The Netherlands | Low back pain | PTs (N = 113), Patients (N = 500) | Outpatient | 1 | Treatment, Management | Yes | Control group:  Adherence: 75/253 (30%)  Non-adherence: 178/253 (70%) |
| French et al. [43] | 2022 | Australia | Low back pain | PTs (N = 182), Chiropractors (N = 88), Patients (N = 1385) | Outpatient | 1 | Treatment, Management | Yes | NA |
| Maas et al. [44] | 2015 | The Netherlands | Upper extremity complaints | PTs (N= 149) | Mixed | 2 | Assessment, Treatment, Management | No | Adherence - Pre-test results:  Intervention group: 474.26 points  Control group: 472.54 points |
| Peter et al. [45] | 2015 | The Netherlands | Hip and knee osteoarthritis | PTs (N = 284) | Mixed | 1 | Assessment, Management | No | Adherence - Baseline results:  Intervention group: 14.4  Control group: 14.5 |
| Peter et al. [46] | 2013 | The Netherlands | Hip and knee osteoarthritis | PTs (N = 203) | Mixed | 1 | Assessment, Treatment, Management | No | Adherence - Baseline results:  Group 1: 56.6  Group 2: 55.6 |
| Schröder et al. [47] | 2023 | Sweden | Low back pain | PTs (N = 98), Patients (N = 388) | Outpatient | 3 | Treatment, Management | Yes | Control group:  Adherence: 42/165 (25%)  Non-adherence: 123/165 (75%) |
| van Dulmen et al. [48] | 2014 | The Netherlands | Low back pain | PTs (N = 90) | NR | 1 | Assessment, Treatment, Management | Yes | Adherence - Baseline score:  Intervention group: 63.7%  Control group: 66.8% |
| ***Non-randomised intervention studies*** | | | | | | | | | |
| Beneciuk et al. [49] | 2022 | USA | Neck pain, low back pain | PTs (N = 27), Patients (N = 497) | Outpatient | 2 | Treatment | No | No baseline data |
| Ferguson et al. [50] | 2010 | UK | Low back pain | PTs (N = 360), Patients (N = 2147) | Outpatient | 1 | Management | Yes | NA |
| Ferguson et al. [51] | 2010 | UK | Low back pain | PTs (N = 360), Patients (N = 2147) | Outpatient | 6 | Assessment, Treatment, Management | Yes | NA |
| Rutten et al. [52] | 2013 | The Netherlands | Low back pain | PTs (N = 24) | Outpatient | 2 | Assessment, Treatment, Management | Yes | Pre-test results:  Adherence: 51.5 |
| Thomas & Mackintosh [53] | 2016 | Australia | Risk of falls | Patients (N = 159) | Inpatient | 2 | Management | Yes | NA |

GP: General practitioner; PT: Physiotherapists; NR: Not reported; NA: Not applicable

1. Ackah M, Boakye H, Yeboah CO, Bello AI. Physiotherapy practice patterns in the management of patients with knee osteoarthritis: A national survey on the use of clinical practice guidelines. Physiother Res Int. 2022:e1964. doi: 10.1002/pri.1964

2. Ahern M, Dean CM, Dear BF, Willcock SM, Hush JM. Management of acute low back pain: the practices and perspectives of primary care clinicians in Australia. Aust J Prim Health. 2020;26(3):256-64. doi: 10.1071/py19152

3. Andersson SF, Bergman S, Henriksson EW, Bremander A. Arthritis management in primary care - A study of physiotherapists' current practice, educational needs and adherence to national guidelines. Musculoskeletal Care. 2017;15(4):333-40. doi: 10.1002/msc.1176

4. Bahns C, Happe L, Thiel C, Kopkow C. Physical therapy for patients with low back pain in Germany: a survey of current practice. BMC Musculoskelet Disord. 2021;22(1):563. doi: 10.1186/s12891-021-04422-2

5. Battista S, Salvioli S, Millotti S, Testa M, Dell’Isola A. Italian physiotherapists’ knowledge of and adherence to osteoarthritis clinical practice guidelines: a cross-sectional study. BMC Musculoskelet Disord. 2021;22(1):380. doi: 10.1186/s12891-021-04250-4

6. Bernhardsson S, Öberg B, Johansson K, Nilsen P, Larsson ME. Clinical practice in line with evidence? A survey among primary care physiotherapists in western Sweden. J Eval Clin Pract. 2015;21(6):1169-77. doi: 10.1111/jep.12380

7. Bishop A, Foster NE, Thomas E, Hay EM. How does the self-reported clinical management of patients with low back pain relate to the attitudes and beliefs of health care practitioners? A survey of UK general practitioners and physiotherapists. Pain. 2008;135(1-2):187-95. doi: 10.1016/j.pain.2007.11.010

8. Caffini G, Battista S, Raschi A, Testa M. Physiotherapists' knowledge of and adherence to evidence-based practice guidelines and recommendations for ankle sprains management: a cross-sectional study. BMC Musculoskelet Disord. 2022;23(1):975. doi: 10.1186/s12891-022-05914-5

9. Demont A, Benaïssa L, Recoque V, Desmeules F, Bourmaud A. Spinal pain patients seeking care in primary care and referred to physiotherapy: A cross-sectional study on patients characteristics, referral information and physiotherapy care offered by general practitioners and physiotherapists in France. PLoS One. 2022;17(9):e0274021. doi: 10.1371/journal.pone.0274021

10. de Souza FS, Ladeira CE, Costa LOP. Adherence to Back Pain Clinical Practice Guidelines by Brazilian Physical Therapists: A Cross-sectional Study. Spine (Phila Pa 1976). 2017;42(21):E1251-e8. doi: 10.1097/brs.0000000000002190

11. Donohue A, McLaughlin C, Crowe M, Horgan F. Clinical guideline adherence by physiotherapists working in acute stroke care. Ir Med J. 2014;107(9):287-9.

12. Hendrick P, Mani R, Bishop A, Milosavljevic S, Schneiders AG. Therapist knowledge, adherence and use of low back pain guidelines to inform clinical decisions--a national survey of manipulative and sports physiotherapists in New Zealand. Man Ther. 2013;18(2):136-42. doi: 10.1016/j.math.2012.09.002

13. Husted M, Rossen CB, Jensen TS, Mikkelsen LR, Rolving N. Adherence to key domains in low back pain guidelines: A cross-sectional study of Danish physiotherapists. Physiother Res Int. 2020;25(4):e1858. doi: 10.1002/pri.1858

14. Keating JL, McKenzie JE, O'Connor DA, French S, Walker BF, Charity M, et al. Providing services for acute low-back pain: A survey of Australian physiotherapists. Man Ther. 2016;22:145-52. doi: 10.1016/j.math.2015.11.005

15. Ladeira CE, Cheng MS, da Silva RA. Clinical Specialization and Adherence to Evidence-Based Practice Guidelines for Low Back Pain Management: A Survey of US Physical Therapists. J Orthop Sports Phys Ther. 2017;47(5):347-58. doi: 10.2519/jospt.2017.6561

16. Ladeira CE, Samuel Cheng M, Hill CJ. Physical therapists' treatment choices for non-specific low back pain in Florida: an electronic survey. J Man Manip Ther. 2015;23(2):109-18. doi: 10.1179/2042618613y.0000000065

17. Leemrijse CJ, Plas GM, Hofhuis H, van den Ende CH. Compliance with the guidelines for acute ankle sprain for physiotherapists is moderate in the Netherlands: an observational study. Aust J Physiother. 2006;52(4):293-9. doi: 10.1016/s0004-9514(06)70010-1

18. Moslem WM, Alrwaily M, Almarwani MM. Adherence to low back pain clinical practice guidelines by Saudi physical therapists: a cross-sectional study. Physiother Theory Pract. 2022;38(7):938-51. doi: 10.1080/09593985.2020.1806420

19. Peter WF, Nelissen RG, Vlieland TP. Guideline recommendations for post-acute postoperative physiotherapy in total hip and knee arthroplasty: are they used in daily clinical practice? Musculoskeletal Care. 2014;12(3):125-31. doi: 10.1002/msc.1067

20. Pisani GK, Carvalho C, Serrão P, Sato TO, Serrão FV. Interventions used by Brazilian physiotherapists in the rehabilitation of patellofemoral pain: A web-based survey. Musculoskelet Sci Pract. 2022;59:102554. doi: 10.1016/j.msksp.2022.102554

21. Riera J, Smythe A, Malliaras P. French physiotherapy management of rotator cuff related shoulder pain: An observational study. Musculoskeletal Care. 2021;19(4):484-94. doi: 10.1002/msc.1545

22. Rutten G, Kremers S, Rutten S, Harting J. A theory-based cross-sectional survey demonstrated the important role of awareness in guideline implementation. J Clin Epidemiol. 2009;62(2):167-76.e1. doi: 10.1016/j.jclinepi.2008.04.004

23. Scheffler B, Schimböck F, Schöler A, Rösner K, Spallek J, Kopkow C. Current physical therapy practice and implementation factors regarding the evidence-based ‘Rehabilitation of Mobility after Stroke (ReMoS)’ guideline in Germany: a cross-sectional online survey. BMC Neurol. 2022;22(1):284. doi: 10.1186/s12883-022-02780-5

24. Spitaels D, Hermens R, Van Assche D, Verschueren S, Luyten F, Vankrunkelsven P. Are physiotherapists adhering to quality indicators for the management of knee osteoarthritis? An observational study. Musculoskelet Sci Pract. 2017;27:112-23. doi: 10.1016/j.math.2016.10.010

25. Childs JD, Fritz JM, Wu SS, Flynn TW, Wainner RS, Robertson EK, et al. Implications of early and guideline adherent physical therapy for low back pain on utilization and costs. BMC Health Serv Res. 2015;15:150. doi: 10.1186/s12913-015-0830-3

26. Fritz JM, Childs JD, Wainner RS, Flynn TW. Primary care referral of patients with low back pain to physical therapy: impact on future health care utilization and costs. Spine (Phila Pa 1976). 2012;37(25):2114-21. doi: 10.1097/BRS.0b013e31825d32f5

27. Horn ME, Brennan GP, George SZ, Harman JS, Bishop MD. Clinical Outcomes, Utilization, and Charges in Persons With Neck Pain Receiving Guideline Adherent Physical Therapy. Eval Health Prof. 2015;39(4):421-34. doi: 10.1177/0163278715583510

28. Jansen MJ, Hendriks EJ, Oostendorp RA, Dekker J, De Bie RA. Quality indicators indicate good adherence to the clinical practice guideline on "Osteoarthritis of the hip and knee" and few prognostic factors influence outcome indicators: a prospective cohort study. Eur J Phys Rehabil Med. 2010;46(3):337-45.

29. Johnston J, Mudge S, Kersten P, Jones A. Physiotherapy Alignment with Guidelines for the Management of Stroke in the Inpatient Setting. New Zealand Journal of Physiotherapy. 2013;41:102-11.

30. Kooijman MK, Swinkels IC, Veenhof C, Spreeuwenberg P, Leemrijse CJ. Physiotherapists' compliance with ankle injury guidelines is different for patients with acute injuries and patients with functional instability: an observational study. J Physiother. 2011;57(1):41-6. doi: 10.1016/s1836-9553(11)70006-6

31. Leerar PJ, Boissonnault W, Domholdt E, Roddey T. Documentation of red flags by physical therapists for patients with low back pain. J Man Manip Ther. 2007;15(1):42-9. doi: 10.1179/106698107791090105

32. Lloyd M, Mackintosh A, Grant C, McManus F, Kelly AM, Karunajeewa H, et al. Evidence-based management of patients with vertigo, dizziness, and imbalance at an Australian metropolitan health service: an observational study of clinical practice. Physiother Theory Pract. 2020;36(7):818-25. doi: 10.1080/09593985.2018.1511020

33. Naylor JM, Gibson K, Mills K, Schabrun SM, Livings R, Dennis S, et al. A snapshot of primary care physiotherapy management of knee osteoarthritis in an Australian setting: does it align with evidence-based guidelines? Physiother Theory Pract. 2022:1-10. doi: 10.1080/09593985.2022.2114816

34. Oostendorp RA, Rutten GM, Dommerholt J, Nijhuis-van der Sanden MW, Harting J. Guideline-based development and practice test of quality indicators for physiotherapy care in patients with neck pain. J Eval Clin Pract. 2013;19(6):1044-53. doi: 10.1111/jep.12025

35. Rebbeck T, Macedo LG, Maher CG. Compliance with clinical guidelines for whiplash improved with a targeted implementation strategy: a prospective cohort study. BMC Health Serv Res. 2013;13:213. doi: 10.1186/1472-6963-13-213

36. Rutten GM, Degen S, Hendriks EJ, Braspenning JC, Harting J, Oostendorp RA. Adherence to Clinical Practice Guidelines for Low Back Pain in Physical Therapy: Do Patients Benefit? Phys Ther. 2010;90(8):1111-22. doi: 10.2522/ptj.20090173

37. Sparkes V. Treatment of low back pain: monitoring clinical practice through audit. Physiotherapy. 2005;91(3):171-7. doi: https://doi.org/10.1016/j.physio.2004.10.007

38. Swinkels IC, van den Ende CH, van den Bosch W, Dekker J, Wimmers RH. Physiotherapy management of low back pain: does practice match the Dutch guidelines? Aust J Physiother. 2005;51(1):35-41. doi: 10.1016/s0004-9514(05)70051-9

39. Tang CY, Pile R, Croft A, Watson NJ. Exploring Physical Therapist Adherence to Clinical Guidelines When Treating Patients With Knee Osteoarthritis in Australia: A Mixed Methods Study. Phys Ther. 2020;100(7):1084-93. doi: 10.1093/ptj/pzaa049

40. van der Wees PJ, Hendriks EJ, Jansen MJ, van Beers H, de Bie RA, Dekker J. Adherence to physiotherapy clinical guideline acute ankle injury and determinants of adherence: a cohort study. BMC Musculoskelet Disord. 2007;8:45. doi: 10.1186/1471-2474-8-45

41. Kolb WH, Bade MJ, Bradberry C. Implementation of clinical practice guidelines for low back pain: A case control cohort study of knowledge translation in a multi-site healthcare organization. J Eval Clin Pract. 2022;28(2):288-302. doi: 10.1111/jep.13633

42. Bekkering GE, Hendriks HJ, van Tulder MW, Knol DL, Hoeijenbos M, Oostendorp RA, et al. Effect on the process of care of an active strategy to implement clinical guidelines on physiotherapy for low back pain: a cluster randomised controlled trial. Qual Saf Health Care. 2005;14(2):107-12. doi: 10.1136/qshc.2003.009357

43. French SD, O'Connor DA, Green SE, Page MJ, Mortimer DS, Turner SL, et al. Improving adherence to acute low back pain guideline recommendations with chiropractors and physiotherapists: the ALIGN cluster randomised controlled trial. Trials. 2022;23(1):142. doi: 10.1186/s13063-022-06053-x

44. Maas MJ, van der Wees PJ, Braam C, Koetsenruijter J, Heerkens YF, van der Vleuten CP, et al. An innovative peer assessment approach to enhance guideline adherence in physical therapy: single-masked, cluster-randomized controlled trial. Phys Ther. 2015;95(4):600-12. doi: 10.2522/ptj.20130469

45. Peter W, van der Wees PJ, Verhoef J, de Jong Z, van Bodegom-Vos L, Hilberdink WK, et al. Effectiveness of an interactive postgraduate educational intervention with patient participation on the adherence to a physiotherapy guideline for hip and knee osteoarthritis: a randomised controlled trial. Disabil Rehabil. 2015;37(3):274-82. doi: 10.3109/09638288.2014.913708

46. Peter WF, van der Wees PJ, Verhoef J, de Jong Z, van Bodegom-Vos L, Hilberdink WK, et al. Postgraduate education to increase adherence to a Dutch physiotherapy practice guideline for hip and knee OA: a randomized controlled trial. Rheumatology (Oxford). 2013;52(2):368-75. doi: 10.1093/rheumatology/kes264

47. Schröder K, Öberg B, Enthoven P, Hedevik H, Abbott A. Improved adherence to clinical guidelines for low back pain after implementation of the BetterBack model of care: A stepped cluster randomized controlled trial within a hybrid type 2 trial. Physiother Theory Pract. 2023;39(7):1376-90. doi: 10.1080/09593985.2022.2040669

48. van Dulmen SA, Maas M, Staal JB, Rutten G, Kiers H, Nijhuis-van der Sanden M, et al. Effectiveness of peer assessment for implementing a Dutch physical therapy low back pain guideline: cluster randomized controlled trial. Phys Ther. 2014;94(10):1396-409. doi: 10.2522/ptj.20130286

49. Beneciuk JM, Osborne R, Hagist MB, Crittenden J, Buzzanca KE, Gao H, et al. American Physical Therapy Association Clinical Practice Guideline Implementation for Neck and Low Back Pain in Outpatient Physical Therapy: A Nonrandomized, Cross-sectional Stepped-Wedge Pilot Study. J Orthop Sports Phys Ther. 2022;52(2):113-23. doi: 10.2519/jospt.2022.10545

50. Ferguson F, Holdsworth L, Rafferty D. Low back pain and physiotherapy use of red flags: the evidence from Scotland. Physiotherapy. 2010;96(4):282-8. doi: 10.1016/j.physio.2010.01.001

51. Ferguson F, Holdsworth L, Rafferty D. A national framework for supporting improvements in the physiotherapy assessment and management of low back pain: the Scottish experience. Physiotherapy. 2010;96(3):198-205. doi: 10.1016/j.physio.2010.02.001

52. Rutten GM, Harting J, Bartholomew LK, Schlief A, Oostendorp RA, de Vries NK. Evaluation of the theory-based Quality Improvement in Physical Therapy (QUIP) programme: a one-group, pre-test post-test pilot study. BMC Health Serv Res. 2013;13:194. doi: 10.1186/1472-6963-13-194

53. Thomas S, Mackintosh S. Improvement of Physical Therapist Assessment of Risk of Falls in the Hospital and Discharge Handover Through an Intervention to Modify Clinical Behavior. Phys Ther. 2016;96(6):764-73. doi: 10.2522/ptj.20150215
