# Supplementary material for: Measuring guideline adherence in physiotherapy: A scoping review of methodological approaches
Source: J Eval Clin Pract. 2024 Oct 27;31(5):10.1111/jep.14218. doi: 10.1111/jep.14218 (PMC12381545; doi:10.1111/jep.14218)
Supplement: Supplementary file 4 — Supporting information. [file JEP-31-0-s001.docx]

**Additional file 4:** Methods for evaluating guideline adherence

| **Study** | **Year** | **Synonym** | **Assessment method** | **Unit of analysis** | **Operationalisation (definition)** | **Operationalisation (quantification)** |
| --- | --- | --- | --- | --- | --- | --- |
| ***Cross-sectional studies*** | | | | | | |
| Ackah et al. [1] | 2022 | NR | Web-based survey  Clinical vignettes (1) | Provider | No operationalisation of guideline adherence.  Only frequency distributions of self-reported use of recommended/not recommended interventions at item level were reported. | |
| Ahern et al. [2] | 2020 | NR | Web-based survey | Provider | No operationalisation of guideline adherence.  Only frequency distributions of self-reported use of recommended interventions at item level were reported. | |
| Andersson et al. [3] | 2017 | Adherence | Web-based survey | Provider | No operationalisation of guideline adherence.  Only frequency distributions of self-reported use of recommended/not recommended interventions at item level were reported. | |
| Bahns et al. [4] | 2021 | Adherence | Web-based survey | Provider | Scoring system (assessment 0-23 points; treatment 0-32 points)  Adherence (within one domain) = PTs scoring ≥ 80% of the maximum possible points  Overall adherence = PTs scoring ≥ 80 % of the maximum possible points within both domains | Binary measure  Proportion of adherent/non-adherent PTs |
| Battista et al. [5] | 2022 | Adherence | Web-based survey  Clinical vignettes (1) | Provider | Delivering = PTs who chose all recommended treatments and none of the non-recommended treatments  Partially delivering = PTs who chose only some of the recommended treatments but none of the non-recommended treatments  Non-delivering = PTs who chose at least one of the non-recommended treatments or who decided either not to treat the patient or to treat the patient for less than five sessions | Multi-categorical measure  Proportion of delivering, partially delivering and non-delivering PTs |
| Bernhardsson et al. [6] | 2015 | NR | Web-based survey | Provider | No operationalisation of guideline adherence.  Only frequency distributions of self-reported use of recommended/not recommended interventions at item level were reported. | |
| Bishop et al. [7] | 2008 | NR | Postal survey  Clinical vignettes (1) | Provider | Adherence was only evaluated for 3 specific recommendations.  Strictly in line = PTs who chose the answer strictly in line with the guidelines  Partly in line = PTs who chose the answer partly in line with the guidelines  Not in line = PTs who chose the answer not in line with the guidelines | Multi-categorical measure  Proportion of PTs strictly in line, broadly in line and not in line with the guidelines |
| Caffini et al. [8] | 2022 | Adherence | Web-based survey  Clinical vignettes (2) | Provider | Vignette 1:  Following = PTs who only chose treatments with a high level of recommendation (Grade A or Level 1)  Partially following = PTs who chose treatments with a high level of recommendation (Grade A or Level 1) and treatments with a lower level of recommendation (Grade B-C or Level 2)  Partially not following = PTs who only chose treatments with a lower level of recommendation (Grade C-D-E-F or Level 2-3-4)  Not following = PTs who chose treatments that are not recommended  Vignette 2:  Adherence is defined slightly differently, see here for details [8] | Multi-categorical measure  Proportion of PTs following, partially following, partially not following and not following guideline recommendations |
| Demont et al. [9] | 2022 | Compliance | Electronic registration forms | Provider | Compliance = PTs who offered at least one intervention that was based on a recommendation of strong or moderate level | Binary measure  Proportion of compliant/non-compliant PTs |
| de Souza et al. [10] | 2017 | Adherence | Web-based survey  Clinical vignettes (6) | Provider | Fully adherent = at least two mandatory interventions were selected for each case  Partially adherent = at least one correct mandatory intervention was selected  Not adherent = none of the mandatory interventions were selected | Multi-categorical measure  Proportion of fully adherent, partially adherent and not adherent PTs |
| Donohue et al. [11] | 2014 | Adherence, Compliance | Postal and web-based survey | Provider | There is an operationalisation of guideline compliance into compliant, sometimes compliant and non-compliant at item level, but no details of the definition are provided in the methods section. | |
| Hendrick et al. [12] | 2013 | Adherence | Web-based survey  Clinical vignettes (1) | Provider | Adherence was only evaluated for 3 specific recommendations.  Strictly in line = PTs who chose the answer strictly in line with the guidelines  Broadly in line = PTs who chose the answer broadly in line with the guidelines  Not in line = PTs who chose the answer not in line with the guidelines  Methods based on Bishop et al. [7] | Multi-categorical measure  Proportion of PTs strictly in line, broadly in line and not in line with the guidelines |
| Husted et al. [13] | 2020 | Adherence | Web-based survey  Clinical vignettes (2) | Provider | Adherence was only evaluated for 3 specific recommendations.  Strictly in line = PTs who chose the answer strictly in line with the guidelines  Partly in line = PTs who chose the answer partly in line with the guidelines  Not in line = PTs who chose the answer not in line with the guidelines  Methods based on Bishop et al. [7] | Multi-categorical measure  Proportion of PTs strictly in line, partly in line and not in line with the guidelines |
| Keating et al. [14] | 2016 | Adherence, Compliance | Web-based survey  Clinical vignettes (5) | Provider | Adherence was only evaluated for 1 specific recommendation.  Vignette 1-4:  Adherence = PTs not ordering any type of X-ray  Vignette 5:  Adherence = PTs ordering some type of x-ray | Binary measure  Proportion of adherent/non-adherent PTs |
| Ladeira et al. [15] | 2017 | Adherence | Web-based survey  Clinical vignettes (4) | Provider | Adherence = PTs selecting all interventions required in each specific vignette (+ additional interventions) | Binary measure  Proportion of adherent/non-adherent PTs |
| Ladeira et al. [16] | 2015 | Adherence | Web-based survey  Clinical vignettes (2) | Provider | Vignette 1:  Adherence = PTs who chose manual therapy and patient education  Non-adherence = PTs did not choose both (manual therapy and patient education) or only included one of these interventions  Vignette 2:  Adherence is defined slightly differently, see here for details [16] | Binary measure  Proportion of adherent/non-adherent PTs |
| Leemrijse et al. [17] | 2006 | Compliance | Web-based survey | Provider | No operationalisation of guideline adherence.  Only frequency distributions of self-reported use of recommended interventions at item level were reported. | |
| Moslem et al. [18] | 2022 | Adherence | Web-based survey  Clinical vignettes (6) | Provider | Adherence = PTs who chose all mandatory interventions (and non-mandatory interventions) | Binary measure  Proportion of adherent/non-adherent PTs |
|  |  |  |  | Action | High adherence = if ≥ 75% of PTs were adherent  Moderate adherence = if ≥ 50 but < 75% of PTs were adherent  Fair adherence = if ≥ 25 but < 50 of PTs were adherent  Low adherence = if < 25% of PTs were adherent | Multi-categorical measure  Proportion of cases with high, moderate, fair and low adherence |
| Peter et al. [19] | 2014 | NR | Web-based survey | Provider | No operationalisation of guideline adherence.  Only frequencies distributions of self-reported use of recommended/not recommended interventions at item level were reported. | |
| Pisani et al. [20] | 2022 | Compliance | Web-based survey | Provider | Scoring system (0-12 points)  High compliance = PTs scoring ≥ 7 points  Low compliance = PTs scoring < 7 points | Binary measure  Proportion of PTs with high/low compliance |
| Riera et al. [21] | 2021 | NR | Web-based survey  Clinical vignettes (1) | Provider | No operationalisation of guideline adherence.  Only frequency distributions of self-reported use of recommended/not recommended interventions at item level were reported. | |
| Rutten et al. [22] | 2009 | Adherence | Postal survey  Clinical vignettes (3)  Quality indicators | Action | Scoring system (0-100%)  A higher score means greater adherence to  recommendations  Methods based on Rutten et al. [23] | Metric measure |
| Scheffler et al. [24] | 2022 | Adherence | Web-based survey | Provider | No operationalisation of guideline adherence.  Only frequency distributions of self-reported use of recommended interventions at item level were reported. | |
|  |  |  |  | Action | Excellent adherence = recommendations chosen by ≥ 80% of PTs | Binary measure  Proportion of recommendations with excellent/low adherence |
| Spitaels et al. [25] | 2017 | Adherence | Web-based survey  Quality indicators | Action | Good adherence = quality indicators chosen by > 80% of PTs  Low adherence = quality indicators chosen by < 50% of PTs | Binary measure  Proportion of quality indicators with good /low adherence |
| ***Cohort studies*** | | | | | | |
| Childs et al. [26] | 2015 | Adherence | Chart review (national database) | Patient | Adherence = patients who have received ≥ 75% active procedures (consistent with guideline recommendations) of total treatment procedures within each episode of care with at least one or more active procedures during each session  Methods based on Fritz et al. [27] | Binary measure  Proportion of adherent/non-adherent treated patients |
| Fritz et al. [27] | 2012 | Adherence | Chart review (national database) | Patient | Adherence = patients who have received ≥ 75% active procedures (consistent with guideline recommendations) of total treatment procedures within each episode of care with at least one or more active procedures at each visit  Methods based on Fritz et al. [28] | Binary measure  Proportion of adherent/non-adherent treated patients |
| Horn et al. [29] | 2015 | Adherence | Chart review (national database) | Patient | Adherence = patients who have received ≥ 75% active procedures (consistent with guideline recommendations) of total treatment procedures within each episode of care  Methods based on Fritz et al. [28] | Binary measure  Proportion of adherent/non-adherent treated patients |
| Jansen et al. [30] | 2010 | Adherence | Standardised electronic recording form  Quality indicators | Action | Good adherence = when positive recommendations were adhered to in more than 90% and negative ones in less than 10% of the study sample | Binary measure  Proportion of quality indicators with good or bad adherence |
| Johnston et al. [31] | 2013 | Alignment | Chart review (clinical patient records) | Action | No operationalisation of guideline adherence.  Only frequency distributions of alignment with guidelines for each management issue were reported. | |
| Kooijman et al. [32] | 2012 | Adherence, Compliance | Chart review (electronic register)  Quality indicators | Patient | Overall adherence = patients who were treated to all quality indicators  Methods based on van der Wees et al. [33] | Binary measure  Proportion of adherent/non-adherent treated patients |
| Leerar et al. [34] | 2007 | Adherence | Chart review (clinical patient records) | Patient | No operationalisation of guideline adherence.  Only frequency distributions of cases for which red flags were documented according to the guidelines were reported. | |
| Lloyd et al. [35] | 2020 | Adherence | Chart review (clinical patient records) | Patient | No operationalisation of guideline adherence.  Only frequency distributions of patients who received recommended/not recommended treatments were reported. | |
| Naylor et al. [36] | 2022 | Alignment | Chart review (clinical patient records) | Patient | No operationalisation of guideline adherence.  Only frequency distributions of patients who received recommended treatments were reported. | |
| Oostendorp et al. [37] | 2013 | Adherence, Compliance | Chart review (clinical patient records)  Quality indicators | Action | Scoring system (0-100%)  A higher score means greater adherence to recommendations | Metric measure |
|  |  |  |  | Patient | Negligible = 0-15%  Low = 16-25%  Weak = 26-35%  Very inadequate = 36-45%  Inadequate = 46-55%  Adequate = 56-65%  Substantial = 66-75%  Good = 76-85%  Very good = 86-95%  Excellent = 96-100% | Multi-categorical measure  Proportion of negligible, low, weak, very inadequate, inadequate, adequate, substantial, good, very good and excellent treated patients |
| Rebbeck et al. [38] | 2013 | Compliance | Chart review (clinical patient records) | Provider | Overall compliance = participants who chose at least three (out of four) of the criteria that are consistent with the guidelines | Binary measure  Proportion of compliant/non-compliant PTs |
| Rutten et al. [39] | 2010 | Adherence | Standardised electronic recording form  Quality indicators | Action | Scoring system (0-100%)  A higher score means greater adherence to  recommendations | Metric measure |
| Sparkes [40] | 2005 | Compliance | Chart review (clinical patient records) | Patient | No operationalisation of guideline adherence.  Only frequency distributions of patients who received recommended treatments were reported. | |
| Swinkels et al. [41] | 2005 | Adherence | Chart review (national database)  Quality indicators | Patient | Adherence = patients treated according to two out of two criteria (consistent with guideline recommendations) | Binary measure  Proportion of adherent/non-adherent treated patients |
|  |  |  |  | Provider | Adherence = patients treated according to two out of two criteria (consistent with guideline recommendations) | Binary measure  Proportion of adherent/non-adherent treated patients per therapist |
| Tang et al. [42] | 2020 | Adherence | Chart review (clinical patient records) | Patient | No operationalisation of guideline adherence.  Only frequency distributions of patients who received recommended treatments were reported. | |
| van der Wees et al. [33] | 2007 | Adherence | Standardised electronic recording form  Quality indicators | Patient | Overall adherence = patients who were treated to all quality indicators | Binary measure  Proportion of adherent/non-adherent treated patients |
| ***Case-control studies*** | | | | | | |
| Kolb et al. [43] | 2022 | Adherence | Chart review (charge codes)  Quality indicators | Provider (clinic) | Scoring system (0-100%)  A higher score means greater adherence to  recommendations  Good adherence = ≥ 75% active charge code (consistent with guideline recommendations) of total charge codes within each month | Metric measure  Binary measure  Proportion of adherent/non-adherent physiotherapy sites |
| ***Randomised controlled trials*** | | | | | | |
| Bekkering et al. [44] | 2005 | Adherence | Standardised electronic recording form | Patient | Overall adherence = patients for whom all recommendations of the guidelines were fulfilled | Binary measure  Proportion of adherent/non-adherent treated patients |
| French et al. [45] | 2022 | Adherence | Questionnaire (mode of delivery not reported)  Clinical vignettes (4) | Provider | No operationalisation of guideline adherence.  Only frequencies distributions of self-reported use of recommended/not recommended interventions at item level were reported. | |
| Maas et al. [46] | 2015 | Adherence | Electronic questionnaire  Clinical vignettes (4)  Quality indicators | Provider | Scoring system (0-737 points)  A higher score means greater adherence to  recommendations | Metric measure |
| Peter et al. [47] | 2015 | Adherence | Electronic questionnaire  Quality indicators | Provider | Scoring system (0-24 points)  A higher score means greater adherence to recommendations  Methods based on Peter et al. [48] | Metric measure |
| Peter et al. [49] | 2013 | Adherence | Electronic questionnaire  Quality indicators | Provider | Scoring system (0-72 points)  A higher score means greater adherence to recommendations  Methods based on Peter et al. [48] | Metric measure |
| Schröder et al. [50] | 2023 | Adherence | Chart review (electronic register)  Paper-pencil recording form  Quality indicators | Patient | Overall adherence = patients had to be treated with all recommendations | Binary measure  Proportion of adherent/non-adherent treated patients |
| van Dulmen et al. [51] | 2014 | Adherence | Electronic questionnaire  Clinical vignettes (4)  Quality indicators | Action | Scoring system (0-100%)  A higher score means greater adherence to recommendations  Methods based on Rutten et al. [23] | Metric measure |
| ***Non-randomised intervention studies*** | | | | | | |
| Beneciuk et al. [52] | 2022 | Concordance | Chart review (clinical patient records) | Patient | Concordant = providing 2 or more recommended interventions  Partially concordant = providing 1 recommended intervention  Non-concordant = not providing any  recommended interventions | Multi-categorical measure  Proportion concordant, partially concordant and non-concordant treated patients |
| Ferguson et al. [53] | 2010 | Compliance | Standardised electronic recording form | Patient | No operationalisation of guideline adherence.  Only frequency distributions of cases for which red flags were documented according to the guidelines were reported. | |
| Ferguson et al. [54] | 2010 | Compliance | Standardised electronic recording form | Patient | No operationalisation of guideline adherence.  Only frequency distributions of cases for which documented assessment and management factors were reported. | |
| Rutten et al. [55] | 2013 | Adherence | Questionnaire (mode of delivery not reported)  Clinical vignettes (4)  Quality indicators | Provider | Scoring system (0-100%)  A higher score means greater adherence to recommendations | Metric measure |
| Thomas & Mackinthosh [56] | 2016 | Adherence | Chart review (clinical patient records)  Quality indicators | Patient | No operationalisation of guideline adherence.  Only frequency distributions of patients who were managed according to the guidelines were reported. | |

PT: Physiotherapist; NR: Not reported

1. Ackah M, Boakye H, Yeboah CO, Bello AI. Physiotherapy practice patterns in the management of patients with knee osteoarthritis: A national survey on the use of clinical practice guidelines. Physiother Res Int. 2022:e1964. doi: 10.1002/pri.1964

2. Ahern M, Dean CM, Dear BF, Willcock SM, Hush JM. Management of acute low back pain: the practices and perspectives of primary care clinicians in Australia. Aust J Prim Health. 2020;26(3):256-64. doi: 10.1071/py19152

3. Andersson SF, Bergman S, Henriksson EW, Bremander A. Arthritis management in primary care - A study of physiotherapists' current practice, educational needs and adherence to national guidelines. Musculoskeletal Care. 2017;15(4):333-40. doi: 10.1002/msc.1176

4. Bahns C, Happe L, Thiel C, Kopkow C. Physical therapy for patients with low back pain in Germany: a survey of current practice. BMC Musculoskelet Disord. 2021;22(1):563. doi: 10.1186/s12891-021-04422-2

5. Battista S, Salvioli S, Millotti S, Testa M, Dell’Isola A. Italian physiotherapists’ knowledge of and adherence to osteoarthritis clinical practice guidelines: a cross-sectional study. BMC Musculoskelet Disord. 2021;22(1):380. doi: 10.1186/s12891-021-04250-4

6. Bernhardsson S, Öberg B, Johansson K, Nilsen P, Larsson ME. Clinical practice in line with evidence? A survey among primary care physiotherapists in western Sweden. J Eval Clin Pract. 2015;21(6):1169-77. doi: 10.1111/jep.12380

7. Bishop A, Foster NE, Thomas E, Hay EM. How does the self-reported clinical management of patients with low back pain relate to the attitudes and beliefs of health care practitioners? A survey of UK general practitioners and physiotherapists. Pain. 2008;135(1-2):187-95. doi: 10.1016/j.pain.2007.11.010

8. Caffini G, Battista S, Raschi A, Testa M. Physiotherapists' knowledge of and adherence to evidence-based practice guidelines and recommendations for ankle sprains management: a cross-sectional study. BMC Musculoskelet Disord. 2022;23(1):975. doi: 10.1186/s12891-022-05914-5

9. Demont A, Benaïssa L, Recoque V, Desmeules F, Bourmaud A. Spinal pain patients seeking care in primary care and referred to physiotherapy: A cross-sectional study on patients characteristics, referral information and physiotherapy care offered by general practitioners and physiotherapists in France. PLoS One. 2022;17(9):e0274021. doi: 10.1371/journal.pone.0274021

10. de Souza FS, Ladeira CE, Costa LOP. Adherence to Back Pain Clinical Practice Guidelines by Brazilian Physical Therapists: A Cross-sectional Study. Spine (Phila Pa 1976). 2017;42(21):E1251-e8. doi: 10.1097/brs.0000000000002190

11. Donohue A, McLaughlin C, Crowe M, Horgan F. Clinical guideline adherence by physiotherapists working in acute stroke care. Ir Med J. 2014;107(9):287-9.

12. Hendrick P, Mani R, Bishop A, Milosavljevic S, Schneiders AG. Therapist knowledge, adherence and use of low back pain guidelines to inform clinical decisions--a national survey of manipulative and sports physiotherapists in New Zealand. Man Ther. 2013;18(2):136-42. doi: 10.1016/j.math.2012.09.002

13. Husted M, Rossen CB, Jensen TS, Mikkelsen LR, Rolving N. Adherence to key domains in low back pain guidelines: A cross-sectional study of Danish physiotherapists. Physiother Res Int. 2020;25(4):e1858. doi: 10.1002/pri.1858

14. Keating JL, McKenzie JE, O'Connor DA, French S, Walker BF, Charity M, et al. Providing services for acute low-back pain: A survey of Australian physiotherapists. Man Ther. 2016;22:145-52. doi: 10.1016/j.math.2015.11.005

15. Ladeira CE, Cheng MS, da Silva RA. Clinical Specialization and Adherence to Evidence-Based Practice Guidelines for Low Back Pain Management: A Survey of US Physical Therapists. J Orthop Sports Phys Ther. 2017;47(5):347-58. doi: 10.2519/jospt.2017.6561

16. Ladeira CE, Samuel Cheng M, Hill CJ. Physical therapists' treatment choices for non-specific low back pain in Florida: an electronic survey. J Man Manip Ther. 2015;23(2):109-18. doi: 10.1179/2042618613y.0000000065

17. Leemrijse CJ, Plas GM, Hofhuis H, van den Ende CH. Compliance with the guidelines for acute ankle sprain for physiotherapists is moderate in the Netherlands: an observational study. Aust J Physiother. 2006;52(4):293-9. doi: 10.1016/s0004-9514(06)70010-1

18. Moslem WM, Alrwaily M, Almarwani MM. Adherence to low back pain clinical practice guidelines by Saudi physical therapists: a cross-sectional study. Physiother Theory Pract. 2022;38(7):938-51. doi: 10.1080/09593985.2020.1806420

19. Peter WF, Nelissen RG, Vlieland TP. Guideline recommendations for post-acute postoperative physiotherapy in total hip and knee arthroplasty: are they used in daily clinical practice? Musculoskeletal Care. 2014;12(3):125-31. doi: 10.1002/msc.1067

20. Pisani GK, Carvalho C, Serrão P, Sato TO, Serrão FV. Interventions used by Brazilian physiotherapists in the rehabilitation of patellofemoral pain: A web-based survey. Musculoskelet Sci Pract. 2022;59:102554. doi: 10.1016/j.msksp.2022.102554

21. Riera J, Smythe A, Malliaras P. French physiotherapy management of rotator cuff related shoulder pain: An observational study. Musculoskeletal Care. 2021;19(4):484-94. doi: 10.1002/msc.1545

22. Rutten G, Kremers S, Rutten S, Harting J. A theory-based cross-sectional survey demonstrated the important role of awareness in guideline implementation. J Clin Epidemiol. 2009;62(2):167-76.e1. doi: 10.1016/j.jclinepi.2008.04.004

23. Rutten GM, Harting J, Rutten ST, Bekkering GE, Kremers SP. Measuring physiotherapists' guideline adherence by means of clinical vignettes: a validation study. J Eval Clin Pract. 2006;12(5):491-500. doi: 10.1111/j.1365-2753.2006.00699.x

24. Scheffler B, Schimböck F, Schöler A, Rösner K, Spallek J, Kopkow C. Current physical therapy practice and implementation factors regarding the evidence-based ‘Rehabilitation of Mobility after Stroke (ReMoS)’ guideline in Germany: a cross-sectional online survey. BMC Neurol. 2022;22(1):284. doi: 10.1186/s12883-022-02780-5

25. Spitaels D, Hermens R, Van Assche D, Verschueren S, Luyten F, Vankrunkelsven P. Are physiotherapists adhering to quality indicators for the management of knee osteoarthritis? An observational study. Musculoskelet Sci Pract. 2017;27:112-23. doi: 10.1016/j.math.2016.10.010

26. Childs JD, Fritz JM, Wu SS, Flynn TW, Wainner RS, Robertson EK, et al. Implications of early and guideline adherent physical therapy for low back pain on utilization and costs. BMC Health Serv Res. 2015;15:150. doi: 10.1186/s12913-015-0830-3

27. Fritz JM, Childs JD, Wainner RS, Flynn TW. Primary care referral of patients with low back pain to physical therapy: impact on future health care utilization and costs. Spine (Phila Pa 1976). 2012;37(25):2114-21. doi: 10.1097/BRS.0b013e31825d32f5

28. Fritz JM, Cleland JA, Brennan GP. Does adherence to the guideline recommendation for active treatments improve the quality of care for patients with acute low back pain delivered by physical therapists? Med Care. 2007;45(10):973-80. doi: 10.1097/MLR.0b013e318070c6cd

29. Horn ME, Brennan GP, George SZ, Harman JS, Bishop MD. Clinical Outcomes, Utilization, and Charges in Persons With Neck Pain Receiving Guideline Adherent Physical Therapy. Eval Health Prof. 2015;39(4):421-34. doi: 10.1177/0163278715583510

30. Jansen MJ, Hendriks EJ, Oostendorp RA, Dekker J, De Bie RA. Quality indicators indicate good adherence to the clinical practice guideline on "Osteoarthritis of the hip and knee" and few prognostic factors influence outcome indicators: a prospective cohort study. Eur J Phys Rehabil Med. 2010;46(3):337-45.

31. Johnston J, Mudge S, Kersten P, Jones A. Physiotherapy Alignment with Guidelines for the Management of Stroke in the Inpatient Setting. New Zealand Journal of Physiotherapy. 2013;41:102-11.

32. Kooijman MK, Swinkels IC, Veenhof C, Spreeuwenberg P, Leemrijse CJ. Physiotherapists' compliance with ankle injury guidelines is different for patients with acute injuries and patients with functional instability: an observational study. J Physiother. 2011;57(1):41-6. doi: 10.1016/s1836-9553(11)70006-6

33. van der Wees PJ, Hendriks EJ, Jansen MJ, van Beers H, de Bie RA, Dekker J. Adherence to physiotherapy clinical guideline acute ankle injury and determinants of adherence: a cohort study. BMC Musculoskelet Disord. 2007;8:45. doi: 10.1186/1471-2474-8-45

34. Leerar PJ, Boissonnault W, Domholdt E, Roddey T. Documentation of red flags by physical therapists for patients with low back pain. J Man Manip Ther. 2007;15(1):42-9. doi: 10.1179/106698107791090105

35. Lloyd M, Mackintosh A, Grant C, McManus F, Kelly AM, Karunajeewa H, et al. Evidence-based management of patients with vertigo, dizziness, and imbalance at an Australian metropolitan health service: an observational study of clinical practice. Physiother Theory Pract. 2020;36(7):818-25. doi: 10.1080/09593985.2018.1511020

36. Naylor JM, Gibson K, Mills K, Schabrun SM, Livings R, Dennis S, et al. A snapshot of primary care physiotherapy management of knee osteoarthritis in an Australian setting: does it align with evidence-based guidelines? Physiother Theory Pract. 2022:1-10. doi: 10.1080/09593985.2022.2114816

37. Oostendorp RA, Rutten GM, Dommerholt J, Nijhuis-van der Sanden MW, Harting J. Guideline-based development and practice test of quality indicators for physiotherapy care in patients with neck pain. J Eval Clin Pract. 2013;19(6):1044-53. doi: 10.1111/jep.12025

38. Rebbeck T, Macedo LG, Maher CG. Compliance with clinical guidelines for whiplash improved with a targeted implementation strategy: a prospective cohort study. BMC Health Serv Res. 2013;13:213. doi: 10.1186/1472-6963-13-213

39. Rutten GM, Degen S, Hendriks EJ, Braspenning JC, Harting J, Oostendorp RA. Adherence to Clinical Practice Guidelines for Low Back Pain in Physical Therapy: Do Patients Benefit? Phys Ther. 2010;90(8):1111-22. doi: 10.2522/ptj.20090173

40. Sparkes V. Treatment of low back pain: monitoring clinical practice through audit. Physiotherapy. 2005;91(3):171-7. doi: https://doi.org/10.1016/j.physio.2004.10.007

41. Swinkels IC, van den Ende CH, van den Bosch W, Dekker J, Wimmers RH. Physiotherapy management of low back pain: does practice match the Dutch guidelines? Aust J Physiother. 2005;51(1):35-41. doi: 10.1016/s0004-9514(05)70051-9

42. Tang CY, Pile R, Croft A, Watson NJ. Exploring Physical Therapist Adherence to Clinical Guidelines When Treating Patients With Knee Osteoarthritis in Australia: A Mixed Methods Study. Phys Ther. 2020;100(7):1084-93. doi: 10.1093/ptj/pzaa049

43. Kolb WH, Bade MJ, Bradberry C. Implementation of clinical practice guidelines for low back pain: A case control cohort study of knowledge translation in a multi-site healthcare organization. J Eval Clin Pract. 2022;28(2):288-302. doi: 10.1111/jep.13633

44. Bekkering GE, Hendriks HJ, van Tulder MW, Knol DL, Hoeijenbos M, Oostendorp RA, et al. Effect on the process of care of an active strategy to implement clinical guidelines on physiotherapy for low back pain: a cluster randomised controlled trial. Qual Saf Health Care. 2005;14(2):107-12. doi: 10.1136/qshc.2003.009357

45. French SD, O'Connor DA, Green SE, Page MJ, Mortimer DS, Turner SL, et al. Improving adherence to acute low back pain guideline recommendations with chiropractors and physiotherapists: the ALIGN cluster randomised controlled trial. Trials. 2022;23(1):142. doi: 10.1186/s13063-022-06053-x

46. Maas MJ, van der Wees PJ, Braam C, Koetsenruijter J, Heerkens YF, van der Vleuten CP, et al. An innovative peer assessment approach to enhance guideline adherence in physical therapy: single-masked, cluster-randomized controlled trial. Phys Ther. 2015;95(4):600-12. doi: 10.2522/ptj.20130469

47. Peter W, van der Wees PJ, Verhoef J, de Jong Z, van Bodegom-Vos L, Hilberdink WK, et al. Effectiveness of an interactive postgraduate educational intervention with patient participation on the adherence to a physiotherapy guideline for hip and knee osteoarthritis: a randomised controlled trial. Disabil Rehabil. 2015;37(3):274-82. doi: 10.3109/09638288.2014.913708

48. Peter W, Wees P, Hendriks E, Bie R, Verhoef J, de jong Z, et al. Quality Indicators for Physiotherapy Care in Hip and Knee Osteoarthritis: Development and Clinimetric Properties. Musculoskeletal care. 2013;11. doi: 10.1002/msc.1041

49. Peter WF, van der Wees PJ, Verhoef J, de Jong Z, van Bodegom-Vos L, Hilberdink WK, et al. Postgraduate education to increase adherence to a Dutch physiotherapy practice guideline for hip and knee OA: a randomized controlled trial. Rheumatology (Oxford). 2013;52(2):368-75. doi: 10.1093/rheumatology/kes264

50. Schröder K, Öberg B, Enthoven P, Hedevik H, Abbott A. Improved adherence to clinical guidelines for low back pain after implementation of the BetterBack model of care: A stepped cluster randomized controlled trial within a hybrid type 2 trial. Physiother Theory Pract. 2023;39(7):1376-90. doi: 10.1080/09593985.2022.2040669

51. van Dulmen SA, Maas M, Staal JB, Rutten G, Kiers H, Nijhuis-van der Sanden M, et al. Effectiveness of peer assessment for implementing a Dutch physical therapy low back pain guideline: cluster randomized controlled trial. Phys Ther. 2014;94(10):1396-409. doi: 10.2522/ptj.20130286

52. Beneciuk JM, Osborne R, Hagist MB, Crittenden J, Buzzanca KE, Gao H, et al. American Physical Therapy Association Clinical Practice Guideline Implementation for Neck and Low Back Pain in Outpatient Physical Therapy: A Nonrandomized, Cross-sectional Stepped-Wedge Pilot Study. J Orthop Sports Phys Ther. 2022;52(2):113-23. doi: 10.2519/jospt.2022.10545

53. Ferguson F, Holdsworth L, Rafferty D. Low back pain and physiotherapy use of red flags: the evidence from Scotland. Physiotherapy. 2010;96(4):282-8. doi: 10.1016/j.physio.2010.01.001

54. Ferguson F, Holdsworth L, Rafferty D. A national framework for supporting improvements in the physiotherapy assessment and management of low back pain: the Scottish experience. Physiotherapy. 2010;96(3):198-205. doi: 10.1016/j.physio.2010.02.001

55. Rutten GM, Harting J, Bartholomew LK, Schlief A, Oostendorp RA, de Vries NK. Evaluation of the theory-based Quality Improvement in Physical Therapy (QUIP) programme: a one-group, pre-test post-test pilot study. BMC Health Serv Res. 2013;13:194. doi: 10.1186/1472-6963-13-194

56. Thomas S, Mackintosh S. Improvement of Physical Therapist Assessment of Risk of Falls in the Hospital and Discharge Handover Through an Intervention to Modify Clinical Behavior. Phys Ther. 2016;96(6):764-73. doi: 10.2522/ptj.20150215
